# Efficacy of Testosterone Replacement Therapy in Correcting Anemia in Men With Hypogonadism: A Randomized Clinical Trial

**DOI:** 10.1001/jamanetworkopen.2023.40030

**Published:** 2023-10-27

**Authors:** Karol M. Pencina, Thomas G. Travison, Andrew S. Artz, A. Michael Lincoff, Steven E. Nissen, Panagiotis Flevaris, Anna Chan, Xue Li, Scott A. Diegel, Kathleen Wannemuehler, Shalender Bhasin

**Affiliations:** 1 Research Program in Men’s Health: Aging and Metabolism, Boston Claude D. Pepper Older Americans Independence Center, Brigham and Women’s Hospital, Harvard Medical School, Boston, Massachusetts; 2 Marcus Institute for Aging Research, Hebrew Senior Life; Division of Gerontology, Beth Israel Deaconess Medical Center, Harvard Medical School, Boston, Massachusetts; 3 Department of Hematology & Hematopoietic Cell Transplantation, City of Hope, Duarte, California; 4 Cleveland Clinic Coordinating Center for Clinical Research (C5Research), Department of Cardiovascular Medicine, Cleveland Clinic, Cleveland, Ohio; 5 AbbVie Inc, North Chicago, Illinois; 6 Department of Biostatistics and Medical Informatics, Statistical Data Analysis Center, University of Wisconsin, Madison

## Abstract

**Importance:**

Testosterone deficiency causes mild anemia. Whether testosterone replacement therapy (TRT) can correct anemia or prevent the development of anemia in men with hypogonadism remains incompletely understood.

**Objective:**

To assess the efficacy of TRT in correcting anemia in men with hypogonadism and anemia, and reducing the risk of developing anemia in those without anemia.

**Design, Setting, and Participants:**

This randomized, placebo-controlled trial included men with hypogonadism at 316 US sites enrolled between May 2018 and February 2022. This study was nested within the Testosterone Replacement Therapy for Assessment of Long-term Vascular Events and Efficacy Response in Hypogonadal Men (TRAVERSE) Study, which evaluated the effect of TRT on major adverse cardiovascular events in middle-aged and older men with hypogonadism. Eligible participants were aged 45 to 80 years, with 2 testosterone concentration results below 300 ng/dL, hypogonadal symptoms, and cardiovascular disease (CVD) or increased CVD risk. The last study visit took place in January 2023. Data were analyzed between March and August 2023.

**Intervention:**

Participants were randomized with stratification for preexisting CVD to 1.62% testosterone gel or placebo gel daily for the study duration.

**Main Outcomes and Measures:**

Proportion of participants with anemia (hemoglobin below 12.7 g/dL) whose anemia remitted (hemoglobin 12.7 g/dL or above) over the study duration. Secondary end points included incidence of anemia among men who were not anemic. Binary end points were analyzed using repeated-measures log-binomial regression.

**Results:**

A total of 5204 men were included, 815 with anemia (mean [SD] age, 64.8 [7.7] years; 247 Black [30.3%], 544 White [66.7%], 24 other [2.9%]) and 4379 without anemia (mean [SD] age, 63.0 [7.9] years; 629 Black [14.4%], 3603 White [82.3%], 147 other [3.4%]). Anemia corrected in a significantly greater proportion of testosterone-treated than placebo-treated men at 6 months (143 of 349 [41.0%] vs 103 of 375 [27.5%]), 12 months (152 of 338 [45.0%] vs 122 of 360 [33.9%]), 24 months (124 of 290 [42.8%] vs 95 of 307 [30.9%]), 36 months (94 of 216 [43.5%] vs 76 of 229 [33.2%]), and 48 months (41 of 92 [44.6%] vs 38 of 97 [39.2%]) (*P* = .002). Among participants without anemia, a significantly smaller proportion of testosterone-treated men developed anemia than placebo-treated men. Changes in hemoglobin were associated with changes in energy level.

**Conclusions and Relevance:**

In middle-aged and older men with hypogonadism and anemia, TRT was more efficacious than placebo in correcting anemia. Among men who were not anemic, a smaller proportion of testosterone-treated men developed anemia than placebo-treated men.

**Trial Registration:**

ClinicalTrials.gov Identifier: NCT03518034

## Key Points

**Question:**

Can testosterone replacement therapy (TRT) correct anemia or prevent the development of anemia in middle-aged and older men with hypogonadism?

**Findings:**

In this randomized clinical trial of 5204 men with hypogonadism, a greater proportion of testosterone-treated men corrected their anemia than placebo-treated men. In men without anemia at baseline, TRT also was associated with a lower incidence of anemia than placebo.

**Meaning:**

These results suggest testosterone treatment is more efficacious than placebo in correcting anemia and preventing the development of anemia in middle-aged and older men with hypogonadism.

## Teaser

This randomized clinical trial examines the effectiveness of testosterone replacement therapy to correct anemia in men with hypogonadism.

## Introduction

Anemia is prevalent in middle-aged and older adults^1,2,3,4,5^ and is associated with impaired quality of life, fatigue, mobility limitation, falls, and increased risk of mortality.^6,7,8,9,10^ Currently, there is no approved therapy for unexplained anemia that occurs during aging.

Testosterone deficiency causes mild normocytic anemia,^11^ and nearly 15% of older men with hypogonadism experience anemia.^12,13^ Testosterone treatment increases hemoglobin.^14,15^ Secondary analyses of a substudy of the Testosterone Trials (129 participants) reported that testosterone replacement therapy (TRT) increases hemoglobin in older men with hypogonadism and was associated with correction of anemia.^12^ However, a large, randomized efficacy trial of TRT in men with hypogonadism and anemia, and with anemia as its primary outcome, has not been conducted. Furthermore, it is unknown whether TRT can prevent the development of anemia in men with hypogonadism.

In 2015, the US Food and Drug Administration directed testosterone manufacturers to conduct a randomized trial to determine whether TRT increases the risk of major adverse cardiovascular events (MACE). To address this regulatory requirement, the Testosterone Replacement Therapy for Assessment of Long-Term Vascular Events and Efficacy Response in Hypogonadal Men (TRAVERSE) Study evaluated the effect of TRT and placebo on MACE.^16^ The design and the MACE results of the TRAVERSE Study have been published previously.^16^ The TRAVERSE Anemia Study, nested within the parent TRAVERSE trial, sought to determine the effects of TRT on correction of anemia in middle-aged and older men with hypogonadism. A secondary aim was to determine the effect of TRT on the development of anemia among participants without anemia at baseline. The study also evaluated whether changes in hemoglobin levels in testosterone-treated men were associated with improvements in energy and cognitive function and whether changes in hematocrit were associated with the risk of MACE and venous thromboembolism (VTE).

## Methods

The TRAVERSE trial’s protocol and eligibility criteria have been published.^16,17^ This randomized, double-masked, parallel group, placebo-controlled study was conducted at 316 trial sites in the US, and enrolled men aged 45 to 80 years who had 2 fasting, morning testosterone concentration results below 300 ng/dL (to convert to nanomoles per liter, multiply by 0.0347) and 1 or more signs or symptoms of hypogonadism, and preexisting cardiovascular disease (CVD) or increased risk of CVD. Men with a contraindication to testosterone treatment (eg, erythrocytosis, history of prostate cancer, prostate specific antigen level above 3 ng/mL [to convert to micrograms per liter, multiply by 1]) were excluded.

Study protocol was approved by the national and local institutional review boards for human participants’ research. An independent data and safety monitoring board oversaw the study’s progress and safety data. All participants provided written informed consent. This report follows the Consolidated Standards of Reporting Trials (CONSORT) reporting guideline for randomized studies.

The analytic sample for the correction of anemia included all randomized participants who had anemia at baseline, defined as hemoglobin level below 12.7 g/dL (to convert to grams per liter, multiply by 10). This threshold was based on established criteria used in previous trials to define anemia.^18^ Men who self-reported a known cause of anemia, use of erythropoietic stimulating agents, hematologic malignancy, or missing hemoglobin value at baseline were excluded from the analyses. No evaluation was performed during screening to determine the cause of anemia. The sample for the analysis of incident anemia included all randomized participants who did not have anemia at baseline.

Participants were randomized using an interactive response technology with stratification for preexisting CVD to either 1.62% transdermal testosterone gel (AbbVie, Inc) or matching placebo gel daily until the study’s completion. Participants were started on 40.5 mg of testosterone gel or placebo gel daily and dose was titrated based on serum testosterone and hematocrit levels, using a prespecified dose-titration plan.^16,17^ To maintain masking, sham adjustments were made in the placebo group.

Complete blood counts and blood chemistries were measured at baseline, months 6 and 12, and then annually for the duration of the study. The Hypogonadism Impact of Symptoms Questionnaire (HIS-Q) with 5 subdomains (sexual, energy, sleep, cognition, and mood), a validated patient-reported measure of symptoms of hypogonadism and response to TRT,^19^ was administered at baseline and months 6, 12, 24, and end of study. As reported,^15,16^ adverse events were classified according to the Medical Dictionary for Regulatory Activities and reported for all randomized patients who received at least 1 dose of the study drug.

### Study Outcomes

The primary outcome of the TRAVERSE Anemia Study was the correction of anemia, defined as an increase in hemoglobin level to 12.7 g/dL or above during the intervention period, among randomized participants who had anemia at baseline. Secondary analyses compared the risk of developing anemia postrandomization in patients who did not have anemia at baseline and the proportions of participants whose hemoglobin increased postrandomization by more than 1.0 g/dL above baseline in those with anemia. Other secondary end points included change from baseline in hemoglobin, hematocrit, red cell counts and indices, and energy and cognitive score, ascertained using the energy and cognition domains of HIS-Q. The time to first occurrence of MACE and time to first VTE event were recorded. The study also determined whether the changes in hemoglobin in testosterone-treated men were associated with improvements in energy and cognition in participants with anemia. Total testosterone, dihydrotestosterone, and estradiol concentrations were analyzed at all available time points.

### Statistical Analyses

The prespecified statistical analysis plan is provided in Supplement 1. The analyses were performed using the intention-to-treat principle and all available participant data at each time point were included in the analyses regardless of treatment adherence. Primary outcome, the relative risk of correction of anemia, was analyzed using repeated-measures log-binomial regression with fixed effects for treatment, visit, treatment-by-visit interaction, and adjusted for preexisting CVD and random per-participant repeated-measures effect. An unstructured covariance matrix was assumed. Estimates of the relative risk of TRT over placebo (together with 95% CIs) and a generalized score statistic *P* value testing the null hypothesis of no difference between TRT and placebo across all time points were derived from the model.

Prespecified subgroup analyses were performed in subgroups categorized by preexisting CVD, baseline testosterone (below 250 ng/dL, 250 ng/dL and above), age (younger than 65 years, 65 years or older) and race (Black or African American, White, and other [including American Indian and Alaskan Native, Asian, Native Hawaiian or other Pacific Islander, and multiple reported races]). Race was identified by self-report, with groups with insufficient numbers for meaningful analysis grouped as other. Prespecified subgroup analyses by race were performed because racial differences in hemoglobin levels, prevalence of hemoglobinopathies, and in pharmacokinetics could contribute to differences in response to study intervention. Specifically, because of the greater prevalence of hemoglobinopathies in Black or African American populations, the rationale for subgroup analysis by race was stronger than for other racial groups.

For all subgroup analyses, models with a 3-way interaction between treatment-visit-subgroup were fit, assuming a compound symmetric covariance matrix and type 1 error was not adjusted for multiplicity; therefore, these *P* values are considered nominal. Post hoc sensitivity analyses in which primary end point was censored at 30 and 365 days after the last treatment day were also conducted. The proportions of men with anemia whose hemoglobin increased by 1.0 g/dL or more above baseline, and analysis of incident anemia were performed in similar manner to primary outcome.

Changes in hemoglobin, hematocrit, red cell counts and indices, and sex hormones were analyzed using linear mixed effects models with fixed effects for treatment, visit, treatment-visit interaction, baseline value, and prior CVD status, and allowing for intraparticipant correlation using an unstructured covariance matrix, unless otherwise noted. An omnibus *F* test was used to evaluate whether there was a significant difference between testosterone and placebo groups across all visits. The association between mean postrandomization change in hemoglobin and average change in energy and cognition scores in testosterone-treated men was analyzed using linear regression, adjusted for preexisting CVD. To evaluate whether change in hematocrit was associated with an increased risk of MACE or VTE in testosterone-treated participants with anemia, time-dependent Cox regression models were fit to time to first MACE or VTE, treating change in hematocrit (measured as a percentage) as a time-dependent covariate, adjusting for baseline hematocrit and preexisting CVD. All hypothesis tests used 2-sided significance α level of .05. Analyses were conducted using SAS version 9.4 (SAS Institute Inc) and R version 4.2.1 (R Project for Statistical Computing).

The TRAVERSE parent study planned to enroll 6000 participants based on the projection that this sample size would accrue 256 primary composite MACE events to rule out a hazard ratio of 1.5 at the 95% (2-sided) upper confidence limit with 90% power. The sample size for the anemia study was not prespecified but defined by the number of men in the TRAVERSE Trial who had anemia at baseline.

## Results

**Figure 1.**
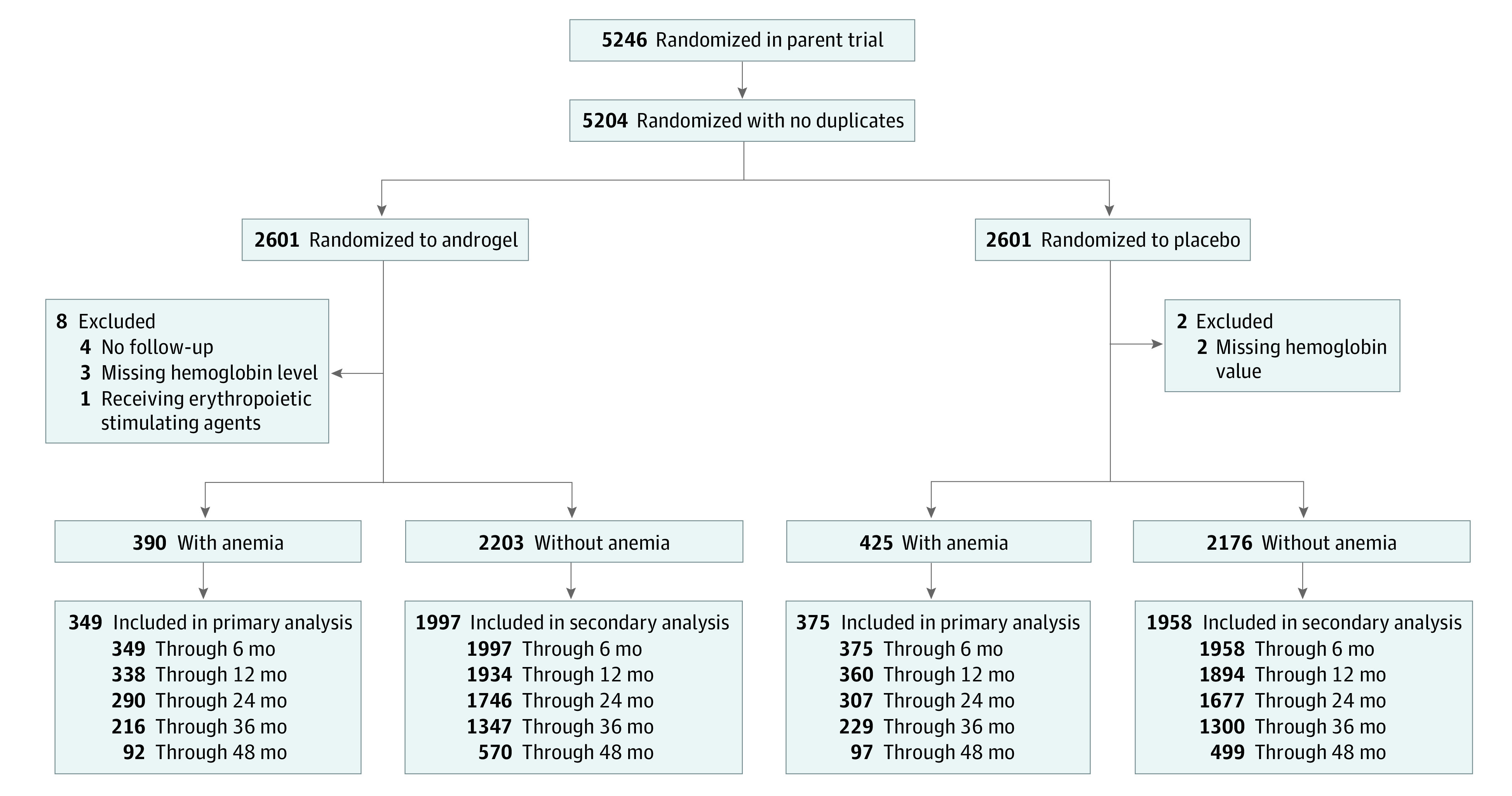
Study Flow Diagram All randomized participants who could be classified as having anemia or not having anemia at baseline and met the eligibility criteria for the parent trial as well as the anemia study were included in the analysis. As this was an event-driven trial and the trial design required the participants to be followed up until the accrual of 256 adjudicated major adverse cardiovascular events. Participants were considered on study if their last visit date in the trial database fell within or after the defined visit window. All randomized participants were included in the primary and secondary analyses if they had at least 1 postbaseline hemoglobin value.

Enrollment took place between May 23, 2018, and February 1, 2022. The last study visit took place on January 19, 2023. As reported previously,^17^ 42 of 5246 enrollments randomized were attributed to 20 participants with duplicate enrollment. After excluding these, the full analysis set included 5204 participants, 2601 in the TRT group and 2603 in placebo group; 815 participants who met eligibility criteria for anemia (390 in TRT and 425 in placebo group) were included in the analysis of correction of anemia (Figure 1). Ten were excluded: 5 had missing baseline hemoglobin, 4 had no follow-up hemoglobin, and 1 person was receiving an erythropoietic stimulating agent. Analysis of incident anemia included 4379 participants without anemia at baseline (2203 in TRT and 2176 in placebo group).

Baseline characteristics were similar between the testosterone and placebo groups in participants with and without anemia (Table). Mean (SD) age of participants with anemia was 64.8 (7.7) years; and their mean (SD) baseline hemoglobin level was 11.8 (0.8) g/dL. By race and ethnicity, 247 participants with anemia were Black (30.3%), 544 White (66.7%), and 24 other (2.9%). Participants who were not anemic at baseline had a mean (SD) age of 63.0 (7.9) years; and their mean (SD) hemoglobin level was 14.4 (1.0) g/dL. This group included 629 Black participants (14.4%), 3603 White (82.3%), and 147 categorized as other (3.4%).

**Table.** Baseline Characteristics of Participants by Treatment Arm and Analysis Population

Variable	Participants, mean (SD) [No. of available participant records]^a^					
	FAS		Anemia at baseline		No anemia at baseline	
	TRT (N = 2601)	Placebo (N = 2603)	TRT (N = 390)	Placebo (N = 425)	TRT (N = 2203)	Placebo (N = 2176)
Age, mean (SD), y	63.3 (7.9)	63.3 (7.9)	64.7 (7.7)	64.9 (7.8)	63.1 (8.0)	62.9 (7.8)
Age group, No. (%)						
45 to <65 y	1360 (52.3)	1392 (53.5)	182 (46.7)	195 (45.9)	1174 (53.3)	1196 (55.0)
≥65 y	1241 (47.7)	1211 (46.5)	208 (53.3)	230 (54.1)	1029 (46.7)	980 (45.0)
Race group, No. (%)						
White	2070 (79.6)	2084 (80.1)	257 (65.9)	287 (67.5)	1808 (82.1)	1795 (82.5)
Black and African American	445 (17.1)	432 (16.6)	119 (30.5)	128 (30.1)	325 (14.8)	304 (14.0)
Other^b^	86 (3.3)	87 (3.3)	14 (3.6)	10 (2.4)	70 (3.2)	77 (3.5)
Ethnicity, No. (%)						
Hispanic and Latino	409 (15.7)	439 (16.9)	43 (11.0)	76 (17.9)	366 (16.6)	362 (16.7)
Not Hispanic or Latino	2191 (84.3)	2162 (83.1)	347 (89.0)	349 (82.1)	1836 (83.4)	1812 (83.3)
Missing	1	2	NA	NA	1	2
Hemoglobin, g/dL	14.0 (1.3) [2595]	14.0 (1.3) [2601]	11.8 (0.8)	11.8 (0.7)	14.4 (1.0)	14.4 (1.0)
Erythrocytes, × 10^12^/L	4.7 (0.5) [2595]	4.7 (0.4) [2601]	4.2 (0.4)	4.2 (0.4)	4.8 (0.4)	4.8 (0.4)
Hematocrit, %	41.9 (3.8) [2595]	41.8 (3.8) [2599]	36.4 (2.4)	36.3 (2.4)	42.9 (3.1)	42.9 (3.0) [2174]
Mean corpuscular volume, fL	89.0 (5.3) [2595]	89.4 (5.3) [2599]	87.2 (6.9)	87.6 (6.6)	89.4 (4.9)	89.8 (5.0) [2174]
Mean corpuscular hemoglobin, pg	29.8 (2.1) [2595]	30.0 (2.0) [2601]	28.5 (2.8)	28.8 (2.5)	30.0 (1.8)	30.2 (1.8)
Mean corpuscular hemoglobin concentration, g/L	335.1 (13.8) [2595]	335.3 (13.7) [2599]	326.6 (15.2)	328.3 (14.5)	336.6 (13.0)	336.7 (13.1) [2174]
Prior CVD	1410 (54.2)	1437 (55.2)	233 (59.7)	261 (61.4)	1175 (53.3)	1175 (54.0)
Current alcohol use						
Yes	1374 (52.9)	1412 (54.4)	179 (45.9)	202 (47.6)	1191 (54.1)	1209 (55.7)
No	1223 (47.1)	1183 (45.6)	211 (54.1)	222 (52.4)	1010 (45.9)	961 (44.3)
Missing	4	8	NA	1	2	6
Current nicotine use						
Yes	527 (20.3)	534 (20.5)	63 (16.2)	74 (17.4)	464 (21.1)	460 (21.1)
No	2072 (79.7)	2068 (79.5)	327 (83.8)	351 (82.6)	1738 (78.9)	1716 (78.9)
Missing	2	1	NA	NA	1	NA
Testosterone, ng/dL	220.5 (47.0) [2596]	220.1 (48.1) [2602]	209.7 (51.6)	210.7 (49.6)	222.4 (45.9)	221.9 (47.6)
Dihydrotestosterone, ng/dL	16.1 (7.9) [2487]	16.2 (8.5) [2498]	14.8 (8.3) [374]	14.9 (8.1) [410]	16.3 (7.8) [2110]	16.5 (8.6) [2087]
Estradiol, pg/mL	21.0 (8.2) [2470]	21.0 (8.4) [2494]	18.9 (8.0) [377]	18.1 (7.7) [409]	21.4 (8.2) [2090]	21.5 (8.4) [2084]
Aspirin use	1570 (60.4)	1546 (59.4)	270 (69.2)	271 (63.8)	1298 (58.9)	1275 (58.6)
Energy domain score (HIS-Q)	50.7 (23.3) [2424]	50.1 (23.1) [2396]	50.8 (23.7) [363]	48.0 (23.5) [388]	50.6 (23.2) [2059]	50.5 (23.0) [2007]
Cognition domain score (HIS-Q)	37.0 (17.2) [2423]	36.0 (16.7) [2397]	37.9 (17.5) [363]	35.8 (16.9) [388]	36.8 (17.1) [2058]	36.1 (16.7) [2008]

Abbreviations: FAS, full analysis set; CVD, cardiovascular disease; HIS-Q, hypogonadism impact of symptoms questionnaire.

SI conversion factors: To convert serum total testosterone concentrations to nanomoles per liter, multiply testosterone concentration in nanograms per deciliter by 0.0347; estradiol concentrations to picomoles per liter, multiply by 3.67; dihydrotestosterone concentrations to nanomoles per liter, multiply by 0.0344.

^a^ Ten randomized participants were excluded from the analysis (see eFigure 1 in Supplement 2).

^b^ Other includes Asian, American Indian and Alaskan Native, multiracial, Native Hawaiian or other Pacific Islander.

Of 815 men with anemia, 803 (98.5%) were followed up for at least 6 months, 768 (94.2%) for 1 year, 653 (80.1%) for 2 years, 484 (59.4%) for 3 years, and 206 (25.3%) for 4 years (eFigure 1 in Supplement 2). Among those with anemia, the mean (SD) treatment duration was 20.7 (14.0) and 20.5 (13.8) months in the TRT and placebo groups, respectively. Total testosterone, DHT, and estradiol levels increased significantly more in TRT compared with placebo in those with and without anemia (eTable 3 in Supplement 2).

### Correction of Anemia

**Figure 2.**
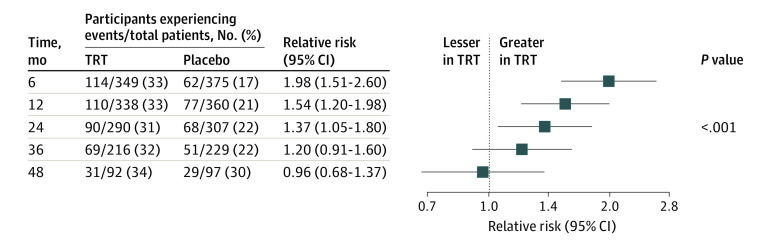
Correction of Anemia Among Participants Who Had Anemia at Baseline Frequencies and relative risks of correction of anemia in the testosterone replacement therapy (TRT) group relative to placebo group and 95% CIs at each visit in men who had anemia at baseline are shown by treatment group and time point. The omnibus test *P* value shown in the figure is a test of the null hypothesis of no difference between TRT and placebo groups across all time points.

**Figure 3.**
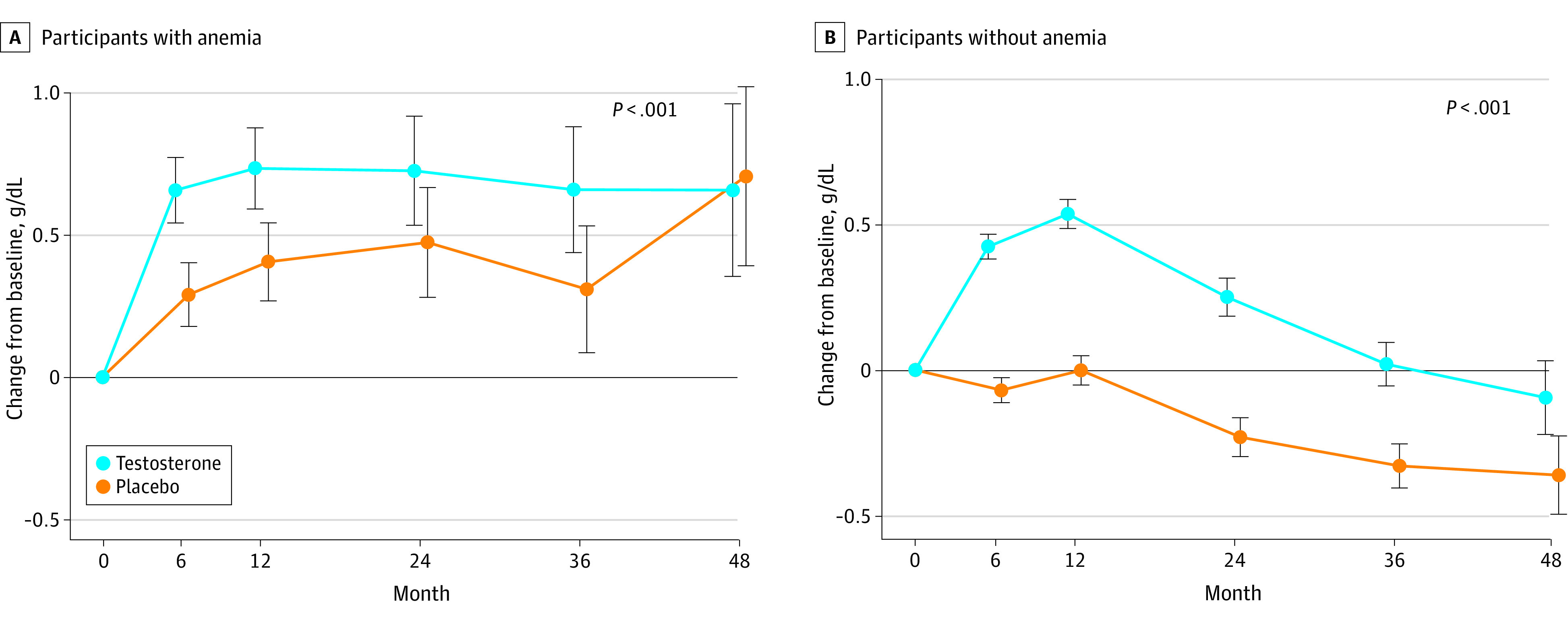
Men With Hemoglobin Increase in Participants Who Had Anemia at Baseline Frequencies and relative risks of hemoglobin increase of more than 1.0 g/dL above baseline in the TRT group relative to the placebo group and the associated 95% CIs at each visit in men who had anemia at baseline are shown by treatment group and time point. The risk ratio of hemoglobin increase of more than 1.0 g/dL in the TRT vs placebo group was estimated by a repeated measures log-binomial regression with fixed effects for treatment, visit, treatment-visit interaction, preexisting cardiovascular disease, and a random per-subject repeated measures effect using an unstructured covariance matrix. The omnibus test *P* value shown in the figure is a test of the null hypothesis of no difference between TRT and placebo groups across all time points.

Participants randomized to testosterone group with anemia were significantly more likely to experience correction of anemia than those randomized to placebo (Figure 2). The proportion of participants whose anemia was corrected was significantly greater in the TRT than the placebo group at 6 months (143 of 349 [41.0%] vs 122 of 360 [27.5%]), 12 months (152 of 338 [45.0%] vs 122 of 360 [33.9%]), 24 months (124 of 290 [42.8%] vs 95 of 307 [30.9%]), 36 months (94 of 216 [43.5%] vs 76 of 229 [33.2%]), and 48 months (41 of 92 [44.6%] vs 38 of 97 [39.2%]) at month 48 (omnibus test *P* = .002) (Figure 2). The proportion of participants whose hemoglobin increased more than 1.0 g/dL during treatment was significantly greater in the TRT than in the placebo group at 6 months (114 of 349 [32.7%] vs 62 of 375 [16.5%]), 12 months (110 of 338 [32.5%] vs 77 of 360 [21.4%]), 24 months (90 of 290 [31.0%] vs 68 of 307 [22.2%]), 36 months (69 of 216 [31.9%] vs 51 of 229 [22.3%]), and 48 months (31 of 92 [33.7%] vs 29 of 97 [29.9%]) (omnibus test *P* < .001) (Figure 3).

Among participants with anemia at baseline, hemoglobin levels increased significantly more in the TRT than placebo group at 6 months (between-group least square mean difference, 0.37 g/dL; 95% CI, 0.21 to 0.53 g/dL), 12 months (0.33 g/dL; 95% CI, 0.13 to 0.53 g/dL), 24 months (0.25 g/dL; 95% CI, −0.02 to 0.53 g/dL), 36 months (0.35 g/dL; 95% CI, 0.04 to 0.67 g/dL), and 48 months (−0.05 g/dL; 95% CI, −0.49 to 0.39 g/dL) (omnibus *P* < .001) (eFigure 1 in Supplement 2). The mean change in hemoglobin level in testosterone-treated participants who had anemia was associated negatively with change in the HIS-Q energy domain score, indicating an improvement in energy level, although the effect size was small—a 1-SD increase in hemoglobin was associated with an −0.14 decrease (SE, 0.055) in energy score (*P* = .01) (eFigure 2 in Supplement 2). There was no significant association between hemoglobin and HIS-Q cognitive domain score.

### Development of Anemia

**Figure 4.**
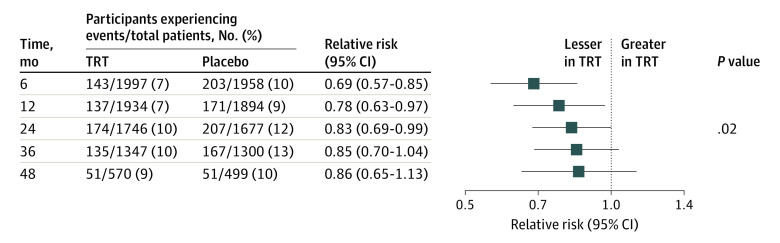
Incidence of Anemia in Participants Who Did Not Have Anemia at Baseline Frequencies and relative risks of incident anemia in the TRT group relative to placebo and 95% CIs at each visit in men who did not have anemia at baseline are shown by treatment group and time point. The risk ratio of incident anemia in the TRT vs placebo group was estimated by a repeated measures log-binomial regression with fixed effects for treatment, visit, treatment-visit interaction, preexisting cardiovascular disease, and a random per-subject repeated measures effect using an unstructured covariance matrix. The omnibus test *P* value shown in the figure is a test of the null hypothesis of no difference between TRT and placebo groups across all time points.

Among participants without anemia, a significantly smaller proportion of participants in the testosterone group developed anemia postrandomization compared with placebo group at 6 months (143 of 1997 [7.2%] vs 203 of 1958 [10.4%]), 12 months (137 of 1934 [7.1%] vs 171 of 1894 [9.0%]), 24 months (174 of 1746 [10.0%] vs 207 of 1677 [12.3%]), 36 months (135 of 1347 [10.0%] vs 167 of 1300 [12.9%]), and 48 months (51 of 570 [9.0%] vs 51 of 499 [10.2%]) (omnibus test *P* = .02) (Figure 4). The increase in hemoglobin among men without anemia was significantly greater in testosterone-treated men than in placebo-treated men at all visits: 6 months (least square mean between-group difference, 0.49 g/dL; 95% CI, 0.43-0.55 g/dL), 12 months (0.54 g/dL; 95% CI, 0.47-0.61 g/dL), 24 months (0.48 g/dL; 95% CI, 0.39-0.58 g/dL), 36 months (0.35 g/dL; 95% CI, 0.24-0.46 g/dL), and 48 months (0.27 g/dL; 95% CI, 0.08-0.45 g/dL) (omnibus *P* < .001).

### Red Cell Indices

Red cell counts and hematocrit levels increased significantly more in testosterone-treated men than in placebo-treated men among those who had anemia as well as in those without anemia at baseline (eTable 1 in Supplement 2). Mean corpuscular volume, mean corpuscular hemoglobin, and mean corpuscular hemoglobin concentration decreased more in testosterone-treated men than placebo-treated men without anemia but did not change significantly in either group of men with anemia (eTable 2 in Supplement 2).

### Prespecified Subgroup Analyses

The findings of prespecified subgroup analyses by preexisting CVD, baseline testosterone (stratified at 250 ng/dL), age (younger than 65 years, 65 years or older), and race (Black or African American, White) of the effect of TRT on the correction of anemia (eFigure 3 in Supplement 2) and incidence of anemia (eFigure 5 in Supplement 2) were similar to those of the primary analysis. The difference in proportion of men with hemoglobin increase more than 1 g/dL between men without and with CVD was significant (*P* = .04) (eFigure 4 in Supplement 2). Post hoc sensitivity analyses in which the end points were censored 30 days and 365 days after treatment discontinuation yielded similar results for the correction of anemia as well as incident anemia (eFigures 6 and 7 in Supplement 2).

### Adverse Events

The adverse events in the TRAVERSE trial have been previously reported.^17^ The frequencies of investigator-reported adverse events by treatment group in men with and without anemia were similar to that in the parent trial with trends toward greater frequency of nonfatal cardiac arrythmias and acute kidney injury in the testosterone-treated men, especially in men with anemia (eTable 4 in Supplement 2). Six participants, whose hematocrit level exceeded 54% at the lowest (20.25 mg) testosterone dose, had their study medication discontinued. Time-dependent Cox hazards models did not show an association between change in hematocrit and the risk of MACE (HR, 0.97; 95% CI, 0.92-1.02) or VTE (HR, 0.94; 95% CI, 0.84-1.05) in testosterone-treated men with anemia.

## Discussion

Anemia is a common health problem among older men that is associated with fatigue, functional limitations, a reduced ability to carry out activities of daily living, increased risk of falls, exacerbation of existing CVD, and increased risk of hospitalizations and mortality.^1,2,7,8,9,10^ Testosterone deficiency is often an overlooked cause of anemia in older men.^12,13,20^ The TRAVERSE Anemia study provides evidence that TRT was significantly more efficacious than placebo in correcting anemia in middle-aged and older men with hypogonadism. These findings were further corroborated in supportive analysis in which TRT was associated with a greater proportion of men with anemia increasing their hemoglobin level by more than 1 g/dL. TRT also was associated with a reduced incidence of anemia in hypogonadal men who were not anemic. The TRAVERSE Anemia study’s findings of testosterone’s efficacy in correcting anemia and in preventing the development of anemia will facilitate a more informed appraisal by clinicians and patients of the potential benefits and risk of TRT in middle-aged and older men with hypogonadism in whom TRT is being considered.

The magnitude of increase in hemoglobin in testosterone-treated men with anemia was not dissimilar from that reported with other erythropoiesis-stimulating agents and inhibitors of hypoxia-inducible factor prolyl hydroxylase that are approved for the treatment of anemia.^21,22^ TRT was also associated with a greater proportion of men improving their hemoglobin by more than 1 g/dL, a level that has been used to evaluate meaningful treatment response in patients with anemia.^23,24^ The association of change in hemoglobin level with change in energy level in the current study is consistent with the previously reported association of increase in hemoglobin with improvements in 6-minute walking distance, fatigue, and aerobic capacity.^12,25^

Testosterone likely increases hemoglobin and red cell number by multiple mechanisms. Testosterone stimulates erythropoietin transcription, increases iron availability for erythropoiesis by suppressing hepcidin transcription, increases the numbers of common myeloid progenitors, and improves red cell survival.^15,26,27,28,29^ Testosterone corrects anemia of inflammation by improving iron availability and by promoting maturation of erythroid precursors.^28^

The TRAVERSE Anemia Study is the largest testosterone trial to date in older men with hypogonadism and anemia in which correction of anemia was a prespecified primary end point. A secondary analysis of 129 men with unexplained anemia in the testosterone trials also found correction of anemia in a greater proportion of testosterone-treated men than placebo-treated men.^12^ To our knowledge, the TRAVERSE Anemia Study is the first to report the effects of TRT on the incidence of anemia in men with hypogonadism.

### Limitations

This study had several limitations. These findings should not be applied to men who are not hypogonadal, women, transgender and gender diverse people, or to men using supraphysiologic doses of testosterone. Although the TRAVERSE trial’s sample size is to our knowledge the largest of any randomized testosterone trials to date, the sample size of the Anemia Study was defined by the number of randomized participants who had anemia at baseline. The cause of anemia in the enrolled participants was not ascertained. As reported,^17^ the rates of study medication discontinuation were high although not dissimilar from those in in hypogonadal men prescribed TRT in clinical practice.^30^ Randomized trials in other chronic symptomatic conditions, such as menopausal women or chronic pain, tend to have high rates of study medication discontinuation.^31,32^ The trial was conducted during the SARS-CoV-2 pandemic, which affected retention. The rates of nonretention were similar in the 2 treatment arms, and drug discontinuation would only bias the results toward null. Furthermore, sensitivity analyses in which the end points were censored 30 days and 365 days after treatment discontinuation yielded similar results (eFigures 6 and 7 in Supplement 2).

Participants had high rates of obesity, diabetes, CAD, and other risk factors for CAD because eligibility criteria were designed to enroll men with CAD or increased risk of CAD in addition to meeting the criteria for hypogonadism. Surveys of men with hypogonadism^33,34^ and men receiving TRT in the US,^35^ and most randomized testosterone trials, including the Testosterone Trials,^36^ have found high rates of obesity, diabetes and other chronic conditions. The prevalence of anemia in study participants (15.7%) was similar to other studies of older men with hypogonadism.^12^

## Conclusions

In middle-aged and older men with hypogonadism and anemia, TRT was more efficacious than placebo in correcting anemia. TRT was also associated with a reduced incidence of anemia among men without anemia at baseline.

## Notes

Supplement 1.
Trial Protocol and Statistical Plan

Supplement 2. **eAppendix.** TRAVERSE Study Sites and Committees **eFigure 1.** Estimated Change From Baseline in Hemoglobin (g/dL) for Participants with Anemia and without Anemia at Baseline **eFigure 2.** Association of the Standardized Average Change From Baseline in Hemoglobin (g/dL) With the Standardized Average Change in HIS-Q Energy Score in Men With Anemia at Baseline Who Were Assigned to the Active (TRT) Study Group **eFigure 3.** Prespecified Subgroup Analyses of the Risk of Correction of Anemia in Participants Who Had Anemia at Baseline **eFigure 4.** Prespecified Subgroup Analyses of the Risk of Increase in Hemoglobin >1g/dL in Participants Who Had Anemia at Baseline **eFigure 5.** Prespecified Subgroup Analyses of the Risk of Incident Anemia in Participants Who Did Not Have Anemia at Baseline **eFigure 6.** Sensitivity Analyses of the Risk of Correction of Anemia Among Participants Who Had Anemia at Baseline **eFigure 7.** Sensitivity Analyses of the Risk of Incident Anemia Among Participants Who Did Not Have Anemia at Baseline **eTable 1.** Change From Baseline in Red Cell Counts and Hematocrit Levels **eTable 2.** Changes in Red Blood Cell Indices **eTable 3.** Changes in Total Testosterone, DHT and Estradiol Levels **eTable 4.** Listing of Investigator-Reported Adverse Events in Men With Anemia and Men Without Anemia

Supplement 3.
Data Sharing Statement
