# Errors in the Figures and Results

**DOI:** 10.1001/jamanetworkopen.2023.55610

**Published:** 2024-01-23

In the Original Investigation titled “Efficacy of Testosterone Replacement Therapy in Correcting Anemia in Men With Hypogonadism: A Randomized Clinical Trial,”^1^ published October 27, 2023, there was an error in the ordering of Figures 2 through 4. Titles, captions, and in-text citations have been updated, including the citation of a new eFigure that has been added to Supplement 2. Additionally, an incorrect subtotal for participants in the placebo group has been fixed in the Results. This article has been corrected.^1^

## References

[1] Pencina K, Travison TG, Artz AS, . Efficacy of testosterone replacement therapy in correcting anemia in men with hypogonadism: a randomized clinical trial. JAMA Netw Open. 2023;6(10):e2340030. doi:10.1001/jamanetworkopen.2023.40030 37889486 PMC10611996

