# Supplementary material for: Efficacy of Testosterone Replacement Therapy in Correcting Anemia in Men With Hypogonadism: A Randomized Clinical Trial
Source: JAMA Netw Open. 2023 Oct 27;6(10):e2340030. doi: 10.1001/jamanetworkopen.2023.40030 (PMC10611996; doi:10.1001/jamanetworkopen.2023.40030)
Supplement: Supplement 1. — Trial Protocol and Statistical Plan [file jamanetwopen-e2340030-s001.pdf]

Statistical Analysis Plan

Study M16-100

The Efficacy of Testosterone Replacement Therapy in Correcting Anemia in Middle-aged and Older Hypogonadal Men (The Anemia Substudy)

Date: January 10, 2022

Version 1.6

Table of Contents

1.0 Introduction ... 6
2.0 Study Background ... 6
2.1 Objective ... 9
2.1.1 Primary Aims: ... 9
2.1.2 Secondary Aims: ... 10
2.1.3 Hypotheses: ... 11
2.2 Study Design ... 13
2.2.1 Study Design and Design Diagram ... 14
2.2.2 Variables Used for Stratification at Randomization ... 15
2.2.3 Eligibility Criteria ... 15
2.3 Outcomes ... 16
2.3.1 Primary outcomes ... 16
2.3.2 Secondary outcomes ... 16
2.4 Statistical Analyses and Power ... 17
2.4.1 Statistical Analyses ... 17
2.5 Interim Analysis ... 19
2.6 Multiplicity Testing Procedures for Type-I Error Control ... 20
2.7 Missing Data ... 20
3.0 Analysis Populations and Important Subgroups ... 20
3.1 Analysis Population ... 20
3.2 Subgroup analyses ... 21
4.0 Analysis Conventions ... 22
4.1 Definition of Baseline ... 22

4.2 Definition of Final Observation 22
4.3 Definition of Visit Windows 22
5.0 Demographics, Baseline Characteristics, Medical History and Study Drug Exposure 23
5.1 Baseline Characteristics 23
5.2 Report of Treatment Exposure and Compliance 23
6.0 Analysis of Endpoints ....**Error! Bookmark not defined.**
7.0 Summary of Changes 24
7.1 Summary of Changes Between the Previous Version and the Current Version 24
7.2 Summary of Changes in Previous Version 24
8.0 References 25
9.0 Partial List of Tables with Schedule of Activities 29
9.1 Schedule of Study Assessments 29
10.0 Efficacy Analysis Time Windows 30
10.1 Visit window for Anemia sub-study 30
Appendix 1. Criteria for the Diagnosis of Common Conditions associated with Anemia 31

List of Abbreviations

ACI	Anemia of Chronic Inflammation
CBC	Complete Blood Count
CKD	Chronic Kidney Disease
CRP	C-Reactive Protein
CV	Cardiovascular
eGFR	Estimated Glomerular Filtration Rate
ESC	Executive Steering Committee
FAS	Full Analysis Set
GFR	Glomerular filtration rate
HIS-Q	Hypogonadism Impact of Symptoms Questionnaire
IDA	Iron Deficiency Anemia
IgA	Immunoglobulin A
IgG	Immunoglobulin G
IgM	Immunoglobulin M
IRT	Interactive Response Technology
LDH	Lactate Dehydrogenase
MACE	Major Adverse Cardiac Event
MCV	Mean Corpuscular Volume
MDRD	Modification of Diet in Renal Disease
MDS	Myelodysplastic Syndromes
SAP	Statistical analysis plan

SAS	Statistical Analysis System
SD	Standard Deviation
SPEP	Serum Protein Electrophoresis
TRAVERSE	Testosterone Replacement Therapy for Assessment of Long-Term Vascular Events and Efficacy Response in Hypogonadal Men Study
TRT	Testosterone Replacement Therapy
UPEP	Urine Protein Electrophoresis
VTE	Venous Thromboembolic Events

1.0 Introduction

This statistical analysis plan supplement (SAP) describes the statistical methods for the analyses of data collected for The Anemia Sub-study of the TRAVERSE Trial (Study M16-100) and provides the analysis plan to guide the statistical programming work. The scope of this SAP is limited to only the Anemia sub-study.

All analyses will be performed using SAS Version 9.3 or later (SAS Institute, Inc., Cary, NC 27513) and/or R version 3.6.0 or later (R Foundation for Statistical Computing, Vienna). The SAP will be signed off before the study database is locked.

2.0 Study Background

Although several different definitions of anemia exist in the literature, anemia is currently defined by low hemoglobin levels below 12.7 g/dL using contemporary assays for hemoglobin measurement (1). Anemia is highly prevalent in older adults affecting nearly 10 to 12% of adults, 65 years or older, or 3 to 4 million persons in the United States alone (2-14). The prevalence of anemia rises with advancing age to 20 to 30% among those who are 85 years or older. In about one third of community-dwelling older adults, a clearly definable cause of anemia is not identified; these individuals meet the definition of the unexplained anemia of aging (1).

Unexplained anemia of aging is characterized by mild to moderate decrease in hemoglobin level (hemoglobin levels typically between 10 g/dL to 12.7 g/dL in men) with normocytic red cell indices. The pathophysiology of unexplained anemia of

aging is complex and multifactorial, and involves dysregulated erythropoiesis, reduced response to erythropoietin, reduced stem or progenitor cell proliferative capacity, inhibitory effect of inflammation, and ineffective erythropoiesis. The role of age-related decline in testosterone levels as a contributor to unexplained anemia of aging is incompletely understood.

Unexplained anemia of aging is associated with adverse health outcomes, including impaired quality of life, fatigue, functional limitations, mobility problems, falls, and increased risk of mortality (15-19). Currently, there is no approved therapy for the unexplained anemia of aging.

Testosterone deficiency due to either the disorders of the testis, pituitary and the hypothalamus or to administration of androgen deprivation therapy is associated with a decrease in hemoglobin and hematocrit (20-23). In observational studies, the age-related decline in testosterone levels has been associated with anemia in older men (19-20). Testosterone treatment increases hemoglobin and hematocrit in both young and older men and women (21-23). Erythrocytosis is the most frequent adverse event associated with testosterone treatment (22, 25-26). The older men are more sensitive to the effects of testosterone and exhibit greater increments in hemoglobin and hematocrit than young men (24).

The mechanisms by which testosterone increases hemoglobin and hematocrit are not fully understood. Testosterone stimulates iron-dependent erythropoiesis (27). Testosterone increases iron availability for erythropoiesis through suppression of hepcidin (27-29). Testosterone also stimulates erythropoietin secretion, but the effects of testosterone treatment on serum erythropoietin levels are transient (24,

27, 29). With continued testosterone treatment, serum erythropoietin levels return towards baseline, but are not suppressed below baseline in spite of increased hemoglobin level (24, 29), suggesting that testosterone treatment alters the set point that regulates the hemoglobin – erythropoietin ratio (29). It is unknown whether testosterone increases the sensitivity of bone marrow erythropoietic progenitors to erythropoietin. Testosterone also appears to have direct effect on the bone marrow hematopoietic progenitor cells (30); it promotes the differentiation of hematopoietic progenitors into common myeloid progenitors. Older adults often suffer from a number of comorbid conditions which are associated with high burden of chronic inflammation, which also may contribute to anemia.

We have shown that testosterone can effectively correct anemia in a mouse model of anemia of inflammation induced by repeated injections of low doses of heat-killed Brucella abortus (31). In this mouse model of anemia of inflammation, testosterone administration reduces ineffective erythropoiesis (31).

Testosterone administration corrects anemia in a preclinical mouse model of aging (32). Although relatively small trials have provided preliminary evidence that testosterone can increase hemoglobin and hematocrit in unexplained anemia of aging (29, 33), a large randomized trial in men with unexplained anemia of aging has not been conducted. The large sample size of the TRAVERSE Trial offers an outstanding opportunity to determine the efficacy of testosterone replacement therapy in correcting unexplained anemia.

In epidemiologic studies, higher levels of hematocrit are associated with increased risk of myocardial infarction, ischemic stroke, and hypertension in both men and

women 34-38). However, this relation between hematocrit and cardiovascular risk is complex and varies with age and sex (34). the data on the relation of hematocrit levels with the risk of venous thromboembolic events are less consistent across studies (39-40). However, none of the previous randomized trials of testosterone treatment has been large enough or long enough to determine whether increases in hematocrit during testosterone treatment are associated with increased risk of myocardial infarction, stroke, or venous thromboembolic events. The TRAVERSE Trial because of its large sample size and rigorous adjudication of MACE, including stroke and venous thromboembolic events offers an outstanding opportunity to address these important questions.

2.1 Objective

Some of the proposed aims, described below, will require analyses of the enrolled participants who are anemic at baseline while other aims will require analyses of other groups of participants.

2.1.1 Primary Aim:

1. The primary aim of the anemia sub-study is to determine the efficacy of testosterone replacement therapy relative to placebo in correcting anemia in middle-aged and older hypogonadal men.

2.1.2 Secondary Aims:

2. To determine whether testosterone replacement therapy in middle-aged and older hypogonadal men with anemia is more efficacious than placebo in increasing the proportion of men whose hemoglobin increases by more than 1.0 g/dL above baseline
3. To determine whether the changes in hemoglobin levels during the intervention are associated with improvements in energy, ascertained using the energy domain of HIS-Q in all randomized participants
4. To determine whether the changes in hemoglobin levels during the intervention are associated with improvements in cognition, ascertained using the cognition domain of HIS-Q in all in all randomized participants
5. To determine whether testosterone treatment, compared to placebo, is associated with greater changes in platelet count, neutrophil, monocyte, and lymphocyte counts
6. To determine whether increases in hemoglobin, RDW and red cell counts during testosterone treatment are associated with an increased risk of MACE relative to placebo
7. To determine whether increases in hemoglobin and red cell counts during testosterone treatment are associated with an increased risk of having a cerebrovascular event or MACE relative to placebo
8. To determine whether increases in hemoglobin and red cell counts during testosterone treatment are associated with increased risk of venous thromboembolic events (VTE)

9. To determine whether increases in platelet counts during testosterone treatment are associated with increased risk of venous thromboembolic events (VTE), MACE or cerebrovascular events.
10. To determine whether increases in total WBC count, or neutrophil or monocyte counts are associated with increased risk of VTE, MACE or cerebrovascular events
11. To characterize the participant-level factors (such as age, race, baseline body weight and body mass index, smoking, baseline hemoglobin, baseline creatinine, baseline testosterone level, and increase from baseline in testosterone level) that are associated with the level of increase in hemoglobin, red cell count, and hematocrit
12. To determine whether testosterone administration, relative to placebo, is associated with a differential risk of developing incident anemia among non-anemic men
13. To determine whether testosterone administration, relative to placebo, is associated with a differential change in serum hemoglobin level among non-anemic men

2.1.3 Hypotheses:

Primary

1. Among hypogonadal men with anemia, testosterone replacement therapy will be associated with a greater likelihood of correction of anemia relative to placebo.

Secondary

2. Compared to placebo, testosterone replacement therapy will be associated with a greater proportion of middle-aged and older hypogonadal men with anemia whose hemoglobin level increases by more than 1.0 g/dL above baseline.
3. Change from baseline in energy score, measured using the HIS-Q, will be associated with change from baseline in hemoglobin levels.
4. Change in cognition score, ascertained using the cognition domain of HIS-Q will be associated with change in hemoglobin levels.
5. Testosterone treatment relative to placebo will be associated with greater increase in the circulating numbers of platelets, total white blood cells, neutrophils and monocytes but not lymphocytes or other circulating white blood cell types.
6. Change in red cell count, RDW and hemoglobin levels during testosterone treatment will be associated with increased risk of MACE.
7. Change in hemoglobin, red cell count, RDW during testosterone treatment will be associated with increased risk of cerebrovascular accident.
8. Change in red cell count and hemoglobin levels, and RDW during testosterone treatment will be associated with increased risk of venous thromboembolic events (VTE).

9. Change in platelet count during testosterone treatment will be associated with increased risk of VTE, MACE, and cerebrovascular events.
10. Change in total WBC count, and neutrophil and monocyte counts will be associated with increased risk of VTE, MACE, and cerebrovascular events.
11. In analyses of all randomized participants, participant level factors such as age, race, baseline body weight and body mass index, smoking, baseline hemoglobin, baseline creatinine, baseline testosterone level, and increase from baseline in testosterone level will be associated with the level of increase above baseline in hemoglobin, hematocrit, and red cell count.
12. Testosterone administration relative to placebo will be associated with lesser risk of incident anemia in participants who are not anemic at baseline.
13. Testosterone administration relative to placebo will be associated with change in hemoglobin levels among individuals who are not anemic at baseline.

2.2 Study Design

The anemia sub-study will be nested within the parent trial. The complete blood counts are being performed in the parent trial for safety monitoring. The data for hemoglobin, hematocrit and red cell indices will be analyzed at the end of the trial for the proposed analyses. Additional serum samples will be stored for biomarker analyses at the end of the trial. All the outcome variables, such as MACE, VTE, and thrombotic stroke, are being collected as a part of the parent trial. Thus, the

anemia substudy will impose no additional burden on the study staff or the participants.

2.2.1 Study Design and Design Diagram

The TRAVERSE parent trial is a Phase 4, randomized, double-blind, placebo-controlled, multicenter study of topical TRT in symptomatic hypogonadal men with increased risk for CV disease. The initial planned study enrollment is approximately 6,000 subjects based on the projected timing when 256 MACE will occur under initial assumptions of the annual event rate, subject accrual rate, and study discontinuation rate. There will be approximately 400 sites in North America and possibly Puerto Rico. An Interactive Response Technology (IRT) system will randomize subjects to receive either topical testosterone or placebo in a 1:1 ratio. Randomization will be stratified by pre-existing CV disease (Yes/No). Titration of testosterone dose will occur in subjects receiving active testosterone, while sham dosage titrations will occur in subjects receiving placebo gel via the non-blinded central IRT system. The Screening Period is up to 50 days prior to first dose of study drug. Once subjects meet all of the eligibility criteria during Screening, they will be randomized (1:1 ratio) to active study drug or placebo and will be followed until the study ends. Importantly, randomized subjects who elect to discontinue study drug will also be followed until the study ends unless the subject withdraws from the study completely (withdrawal of informed consent). Subjects who discontinue study drug will still be asked to follow their regularly scheduled protocol visits. Subjects who interrupt study drug will be allowed to restart study drug at any time.

The aim of this anemia sub-study is to determine the benefits of testosterone intervention on correcting anemia of aging in middle-aged men, and whether the benefits in older men can be seen with respect to CV occurrence. Furthermore, anemia sub-study will determine whether the changes in hemoglobin levels during the intervention are associated with improvements in energy, ascertained using the energy domain of HIS-Q in all randomized participants.

2.2.2 Variables Used for Stratification at Randomization

Randomization will descend from the parent trial; no additional randomization or stratification will be imposed in this sub-study. In the parent trial, randomization will be stratified by pre-existing CV disease (Yes/No). It is expected that in the parent trial at least 30% of the randomized subjects will satisfy inclusion criteria for pre-existing CV disease criteria (secondary prevention), and the remaining 70% will satisfy CV risk factors criteria (primary prevention) combined. Analyses in this PDD sub-study will acknowledge stratified randomization.

2.2.3 Eligibility Criteria

All participants enrolled in the parent TRAVERSE trial will be eligible for analyses supporting one or more of the Aims listed above. Participants will be restricted from inclusion in specific analyses on the basis of Aim-specific exclusion criteria given below.

Exclusion Criteria for analyses supporting Aims 1 and 2:

- Hemoglobin at least 12.7 g/dL

- Baseline use of erythropoietic stimulating agents, such as erythropoietin, if known

Exclusion criteria for analyses supporting Aims 12 and 13

- Hemoglobin less than 12.7 g/dL (i.e. participants non-anemic at baseline)

Analyses in Aims 3-11 will be performed on the entire parent trial population.

2.3 Outcomes

2.3.1 Primary outcome

- Correction of anemia, defined as blood hemoglobin levels at least 12.7 g/dL. This endpoint can vary by study visit.

2.3.2 Secondary outcomes

Unless otherwise noted, each outcome can vary by study visit

- Treatment response, as indicated by increase in blood hemoglobin from baseline by more than 1 g/dL at any time.
- Continuous hemoglobin levels following randomization
- Cumulative likelihood of correction of anemia at each timepoint
- Cumulative likelihood of treatment response at each timepoint
- Change in the energy domain score of HIS-Q
- Change in the cognition domain score of HIS-Q

- Change from baseline in platelet count, total white blood cell count, and the numbers of circulating neutrophils, monocytes, and lymphocytes
- Adjudicated MACE, including myocardial infarction and thrombotic stroke, and venous thromboembolic events (VTE)
- Cerebrovascular accident
- VTE
- Cumulative likelihood of incident anemia by time

2.3.3 Other measurements

As dictated by availability, pretreatment laboratory results, including red blood cell indices and other components of the complete blood count (CBC) may be employed to characterize participants and their anemia status. Stored serum may be used to perform the following analyses at the end of the trial in men who are deemed anemic, if possible: serum iron, total iron binding capacity, serum ferritin; B12 and folate levels; serum creatinine and estimated GFR using MDRD; LDH and haptoglobin level; CRP, inflammatory cytokines; SPEP/UPEP. Description of these analyses are provided in Appendix 1.

2.4 Statistical Analyses and Power

2.4.1 Statistical Analyses

The primary and secondary hypotheses in the anemia sub-study will be addressed using intention to treat principles. Descriptive characteristics will be summarized by

each treatment group. Summary statistics (N, mean, SD, median, quartile range, minimum and maximum) will be provided for continuous variables and the number and percentage of subjects within each category will be presented for categorical data. Exploratory analyses will assess the pattern of change in endpoints and functional form of association between calendar time, events, and continuous measures. Following this, formal analysis will proceed as described below. All statistical estimates will be accompanied by 95% confidence intervals. Hypothesis testing will be conducted at the 0.05 level.

Longitudinal models will compare risk of incident remission of anemia at all timepoints using a mixed model repeated measures (MMRM) analysis. Estimation of the risk of remission in testosterone relative to placebo will be obtained using log-binomial regression (Bernoulli variance function with log link) and robust variance estimation for standard errors and confidence intervals. If this model fails to converge, the modified Poisson (Poisson likelihood equation with robust variance estimator) will be employed as fit by Generalized Estimating Equations (GEE).

Baseline measurements will be incorporated as outcome measures in a model that includes effects for visit and visit by treatment interaction terms, where the latter quantify the effects of interest at each measurement. Linearity will not be assumed for the effect of time unless this is consistent with exploratory analysis (above). Models will also control for stratification factors. The treatment effect will be estimated by relative risk and risk differences derived from the interaction terms, along with accompanying confidence intervals, at each measurement of endpoints.

Statistical significance will be evaluated using an omnibus test of difference between testosterone and placebo groups at all measurement points simultaneously.

Analyses of **secondary aims and outcomes** will proceed in parallel fashion. For continuous endpoints (e.g. HIS-Q subscores and blood biomarker measures), a normal likelihood equation and identify link function will be employed in estimation. For binary endpoints (e.g. MACE, VTE), an approach identical to that described for the primary outcome will be used.

For the purposes of estimating the relative likelihood of correction of anemia and relative likelihood of treatment response at any time point, a discrete-time survival analysis (e.g. proportional hazards model) will be employed. For this analysis, individuals achieving correction of anemia or treatment response at a given visit will no longer be considered at risk of that outcome going forward.

For analyses of incident anemia (conducted among non-anemic participants in support of Aims 12 and 13), methods paralleling those described above for analyses of correction of anemia will be employed.

For other analyses characterizing individuals according to biomarker of anemia, subgroup analysis and regression models contrasting participant subgroups may be employed.

2.5 Interim Analysis

No interim analysis is planned for this sub-study.

2.6 Multiplicity Testing Procedures for Type-I Error Control

Type-I error adjustments for multiple comparisons are not planned for efficacy endpoints, subgroup analyses, supportive analyses or sensitivity analyses for this sub-study.

2.7 Missing Data

We have no intention to impute data in this sub-study.

If required (e.g. by referees in publication), multiple imputation of endpoints and covariates by the MICE methodology for the report of anemia sub-study results may be considered where appropriate (i.e. for data reported in the manuscripts). This method is notable for being able to handle clustering of repeated measures at the participant level, a feature of the design of this trial and sub-study. Under such circumstances we would consider whether to impute data for all sub-studies simultaneously.

3.0 Analysis Populations and Important Subgroups

3.1 Analysis Population

The Analysis Set pre-specified for anemia sub-study will be a subset of the FAS (Full Analysis Set) comprising all randomized subjects in the main CV study. Eligible subjects from the main study who satisfy the criteria for anemia sub-study will be included in the analysis. The FAS for the analysis of anemia sub-study will include subjects with anemia at baseline.

Subjects will be categorized according to treatment assigned at randomization. The EAS will be mainly used for the summary of subjects' disposition and summary of subjects' demographics and baseline characteristics for the sub-study.

Statistical analyses of treatment effect over time will comprise all subjects from the sub-study, who have baseline and at least one post-randomization measurement.

A modified analysis set for participants censored at any time (and subsequently) that they are deemed treatment noncompliant per the parent trial protocol1, will be also considered and used for sensitivity analyses of efficacy endpoints.

Data from eligible measurements will be included, where eligible is defined as being obtained from questionnaires or scores for which at least 80% of items are non-missing.

3.2 Subgroup analyses

The following subgroup analysis will be carried out for the main study and may be also considered for anemia efficacy sub-study:

- by race
- by age (< 65 year, ≥ 65 years)
- by prior CV disease status (yes, no)
- by baseline total testosterone levels (< 250 mg/dl, ≥ 250 mg/dl)

To assess potential heterogeneity of effects in hemoglobin changes over time additional sub-group analyses might be considered:

- MCV (mean corpuscular volume) levels: < 100 fL or >= 100 fL,

- MCV < 70 fL
- MCV < 70 fL and red blood cell count 4.35 million per microliter or greater CRP levels: < 10 mg/L or >= 10 mg/L,
- GFR levels: < 30 ml/min or >= 30 ml/min
- RDW levels below or above median

4.0 Analysis Conventions

4.1 Definition of Baseline

Baseline on each outcome measure will be defined as the last available measurement obtained prior to the first dose of study drug (defined as on or before Day 1) (Protocol Appendix C).

4.2 Definition of Final Observation

Final observation for each analysis will be the final assessment affiliated with the relevant visit. For example, for twelve-month assessment of hemoglobin levels, the final observation of each relevant measurement affiliated with the 12-month visit will be included in analyses.

4.3 Definition of Visit Windows

Definitions of the visit windows (baseline and on-treatment) are presented in Section 10.0 (Tables 10.1) and schedule of sub-study activities is presented in Section 9.0. These will be harmonized to those of the parent trial, and decisions made for the parent trial will be considered controlling where they deviate from those described here.

5.0 Demographics, Baseline Characteristics, Medical History and Study Drug Exposure

5.1 Baseline Characteristics

Data collected in this sub-study will be documented using summary tables. Statistics for continuous variables will include mean, median, standard deviation, minimum, maximum, and sample size for each treatment group, and two-sided 95% confidence intervals of the mean difference between the treatment groups. Binary variables will be described with frequencies, percentages, and two-sided 95% confidence intervals of the difference in percentages between treatments.

Medical history will mirror the presentation in the parent TRAVERSE trial, and will include at minimum history of depression, CV history; nicotine and alcohol use; and testosterone use.

5.2 Report of Treatment Exposure and Compliance

Continuous summaries of subjects' total duration of treatment with study drug, among participants recruited in anemia sub-study, will be provided for analyzed time intervals.

Total patient-month of exposure will be calculated by summing the duration of treatment (separately for 1- and 2-year follow-up) for all subjects in the analysis set and dividing this sum by 365.25 (= 1 year). In addition, the number and percentage of subjects exposed to study drug will be summarized for the following categories

of exposure duration: ≤ 1 month, 1 to 3 months, 3 to 6 months, 6 months to 1 year, 1 to 2 years, etc.

Study drug compliance will be computed for anemia sub-study participants, separately for all relevant intervals.

7.0 Summary of Changes

7.1 Summary of Changes Between the Previous Version and the Current Version

Not applicable.

7.2 Summary of Changes in Previous Version

Not applicable

8.0 References

1. Beutler E, Waalen J. The definition of anemia: what is the lower limit of normal of the blood hemoglobin concentration? *Blood.* Mar 1 2006;107(5):1747-1750.
2. Izaks GJ, Westendorp RG, Knook DL. The definition of anemia in older persons. *Jama.* May 12 1999;281(18):1714-1717.
3. Merchant AA, Roy CN. Not so benign haematology: anaemia of the elderly. *Br J Haematol.* Jan 2012;156(2):173-185.
4. Salive ME, Cornoni-Huntley J, Guralnik JM, et al. Anemia and hemoglobin levels in older persons: relationship with age, gender, and health status. *J Am Geriatr Soc.* May 1992;40(5):489-496.
5. Chaves PH, Xue QL, Guralnik JM, et al. What constitutes normal hemoglobin concentration in community-dwelling disabled older women? J Am Geriatr Soc 2004;52:1811-6.
6. Guralnik JM, Eisenstaedt RS, Ferrucci L, et al. Prevalence of anemia in persons 65 years and older in the United States: evidence for a high rate of unexplained anemia. Blood 2004;104(8):2263---8.
7. Ania BJ, Suman VJ, Fairbanks VF, et al. Incidence of anemia in older people: an epidemiologic study in a well-defined population. J Am Geriatr Soc 1997;45(7):825-31.
8. Artz AS, Fergusson D, Drinka PJ, et al. Mechanisms of unexplained anemia in the nursing home. J Am Geriatr Soc 2004;52(3):423-7.
9. Ble A, Fink JC, Woodman RC, et al. Renal function, erythropoietin, and anemia of older persons: the InCHIANTI study. Arch Intern Med 2005;165(19):2222-7.
10. Joosten E, Pelemans W, Hiele M, et al. Prevalence and causes of anemia in a geriatric hospitalized population. Gerontology 1992;38:111-7.
11. Nilsson-Ehle H, Jagenburg R, Landahl S, et al. Hematological abnormalities and reference intervals in the elderly. A cross-sectional comparative study of three urban Swedish population samples aged 70, 75 and 81 years. Acta Med Scand 1988;224:595-604.

12. Robinson B, Artz AS, Culleton B, et al. Prevalence of anemia in the nursing home: contribution of chronic kidney disease. J Am Geriatr Soc 2007;55:1566-70.
13. Steensma DP, Tefferi A**.** Anemia in the elderly: how should we define it, when does it matter, and what can be done? Mayo Clin Proc. 2007;82:958-66.
14. Thein M, Ershler WB, Artz AS, et al. Diminished quality of life and physical function in community dwelling elderly with anemia. Medicine (Baltimore) 2009;88:107-14.
15. Chaves PH, Ashar B, Guralnik JM, et al. Looking at the relationship between hemoglobin concentration and prevalent mobility difficulty in older women. Should the criteria currently used to define anemia in older people be reevaluated? J Am Geriatr Soc 2002;50:1257-64.
16. Penninx BW, Guralnik JM, Onder G, et al. Anemia and decline in physical performance among older persons. Am J Med 2003;115:104-10.
17. Penninx BW, Pluijm SM, Lips P, et al. et al. Late life anemia is associated with increased risk of recurrent falls. J Am Geriatr Soc 2005;53:2106-11.
18. Penninx BW, Pahor M, Woodman RC, et al. Anemia in old age is associated with increased mortality and hospitalization. J Gerontol A Biol Sci Med Sci 2006;61:474-9.
19. Makipour S, Kanapuru B, Ershler WB**.** Unexplained anemia in the elderly. Semin Hematol. 2008;45:250-4.
20. Ferrucci L, Maggio M, Bandinelli S, et al. Low testosterone levels and the risk of anemia in older men and women. Arch Intern Med 2006;166:1380-8
21. Liverman, CT, Blazer, DG. Testosterone and aging: clinical research directions. Joseph Henry Press, 2004.
22. Bhasin S, Cunningham GR, Hayes FJ, Matsumoto AM, Snyder PJ, Swerdloff RS, Montori VM; Task Force, Endocrine Society. Testosterone therapy in men with androgen deficiency syndromes: an Endocrine Society clinical practice guideline. J Clin Endocrinol Metab 2010;95:2536-59.
23. Snyder PJ, Bhasin S, Cunningham GR, et al. Effects of Testosterone Treatment in Older Men. N Engl J Med. 2016;374:611-624.

24. Coviello AD, Kaplan B, Lakshman KM, Chen T, Singh AB, Bhasin S 2008 Effects of Graded Doses of Testosterone on Erythropoiesis in Healthy Young and Older Men. J Clin Endocrinol Metab 2008;93:914-919.
25. Calof OM, Singh AB, Lee ML, Kenny AM, Urban RJ, Tenover JL, Bhasin S. Adverse events associated with testosterone replacement in middle-aged and older men: a meta-analysis of randomized, placebo-controlled trials. J Gerontol A Biol Sci Med Sci 2005;60:1451-7.
26. Ponce OJ, Spencer-Bonilla G, Alvarez-Villalobos N, Serrano V, Singh-Ospina N, Rodriguez-Gutierrez R, Salcido-Montenegro A, Benkhadra R, Prokop LJ, Bhasin S, Brito JP. The efficacy and adverse events of testosterone replacement therapy in hypogonadal men: A systematic review and meta-analysis of randomized, placebo-controlled trials. J Clin Endocrinol Metab. 2018 Mar 17. doi: 10.1210/jc.2018-00404. [Epub ahead of print]
27. Guo W, Bachman E, Li M, Roy CN, Blusztajn J, Wong S, Chan SY, Serra C, Jasuja R, Travison TG, Muckenthaler MU, Nemeth E, Bhasin S. Testosterone administration inhibits hepcidin transcription and is associated with increased iron incorporation into red blood cells. Aging Cell. 2013 Apr;12(2):280-91.
28. Bachman E, Feng R, Travison T, Li M, Olbina G, Ostland V, Ulloor J, Zhang A, Basaria S, Ganz T, Westerman M, Bhasin S. Testosterone suppresses hepcidin in men: a potential mechanism for testosterone-induced erythrocytosis. J Clin Endocrinol Metab. 2010 Oct;95(10):4743-7.
29. Bachman E, Travison TG, Basaria S, et al. Testosterone induces erythrocytosis via increased erythropoietin and suppressed hepcidin: evidence for a new erythropoietin/hemoglobin set point. Journals of Gerontology. Series A, Biological Sciences and Medical Sciences. 2014;69(6):725-735.
30. Mirand EA, Gordon AS, Wenig J. Mechanism of testosterone action in erythropoiesis. Nature. 1965;206(981):270-2.
31. Guo W, Schmidt PJ, Fleming MD, Bhasin S. Effects of Testosterone on Erythropoiesis in a Female Mouse Model of Anemia of Inflammation. Endocrinology. 2016;157:2937-46.
32. Guo W, Li M, Bhasin S. Testosterone supplementation improves anemia in aging male mice. J Gerontol A Biol Sci Med Sci. 2014;69(5):505-13.

33. Roy CN, Snyder PJ, Stephens-Shields AJ, Artz AS, Bhasin S, Cohen HJ, Farrar JT, Gill TM, Zeldow B, Cella D, Barrett-Connor E, Cauley JA, Crandall JP, Cunningham GR, Ensrud KE, Lewis CE, Matsumoto AM, Molitch ME, Pahor M, Swerdloff RS, Cifelli D, Hou X, Resnick SM, Walston JD, Anton S, Basaria S, Diem SJ, Wang C, Schrier SL, Ellenberg SS. Association of Testosterone Levels With Anemia in Older Men: A Controlled Clinical Trial. JAMA Intern Med. 2017 Apr 1;177(4):480-490.
34. Gagnon DR, Zhang TJ, Brand FN, Kannel WB. Hematocrit and the risk of cardiovascular disease--the Framingham study: a 34-year follow-up. Am Heart J. 1994 Mar;127(3):674-82.
35. Jin YZ, Zheng DH, Duan ZY, Lin YZ, Zhang XY, Wang JR, Han S, Wang GF, Zhang YJ. Relationship Between Hematocrit Level and Cardiovascular Risk Factors in a Community-Based Population. J Clin Lab Anal. 2015 Jul;29(4):289-93.
36. Kiyohara Y, Ueda K, Hasuo Y, Fujii I, Yanai T, Wada J, Kawano H, Shikata T, Omae T, Fujishima M. Hematocrit as a risk factor of cerebral infarction: long-term prospective population survey in a Japanese rural community. Stroke. 1986 Jul-Aug;17(4):687-92.
37. Brown DW, Giles WH, Croft JB. Hematocrit and the risk of coronary heart disease mortality. Am Heart J. 2001 Oct;142(4):657-63.
38. Panwar B, Judd SE, Warnock DG, McClellan WM, Booth JN 3rd, Muntner P, Gutiérrez OM. Hemoglobin Concentration and Risk of Incident Stroke in Community-Living Adults. Stroke. 2016 Aug;47(8):2017-24
39. Hultcrantz M, Modlitba A, Vasan SK, Sjölander A, Rostgaard K, Landgren O, Hjalgrim H, Ullum H, Erikstrup C, Kristinsson SY, Edgren G. Hemoglobin concentration and risk of arterial and venous thrombosis in 1.5 million Swedish and Danish blood donors. Thromb Res. 2020 Feb;186:86-92.
40. Schreijer AJ, Reitsma PH, Cannegieter SC. High hematocrit as a risk factor for venous thrombosis. Cause or innocent bystander? Haematologica. 2010 Feb;95(2):182-4.

9.0 Partial List of Tables with Schedule of Activities

9.1 Schedule of Study Assessments

Assessment	Screening	Baseline	6 months	12 months	24 months	36 months	48 months	60 months
Complete blood count as in the parent trial	X	X	X	X	X	X	X	X
Blood stored for biomarker analyses*		X		X		X		X
His-Q energy and cognition domain**		X	X	X	X	X	X	X

* If available

**HIS-Q questionnaire is being administered at these time points as a part of the Anemia Substudy. MACE and thromboembolic events are being recorded as they occur, as part of the parent trial.

10.0 Efficacy Analysis Time Windows

10.1 Visit window for Anemia sub-study

Scheduled Visit	Nominal Day<br><br>(Study Day)	Time Window<br><br>(Study Days Range)
Day 1	1	≤ 1
M6	182	92 - 273
M12	364	274 - 546
M24	728	547 - 910
M36	1092	911 - 1274
M48	1456	1275 - 1638
M60	1820	1639 – 2180
Final Visit		2 to ≤ 2 days after the last dose of study drug

Appendix 1. Description of potential biomarker characterizations of anemia

For the purposes of this substudy, participants are classified as anemic if meeting the basic criterion of blood hemoglobin less than 12.7 g/dL. Depending on the availability of additional biomarkers, other analyses may employ characterization of participants by cause of anemia according to the following specification. It is acknowledged that these categories may result in overlapping participant subgroups, which will be acknowledged in analysis and any publication.

Anemic participants may be classified as having a known cause if they had a serum creatinine level of 2.2 mg/dL (to convert to μmol/L multiply by 76.25) or higher (renal insufficiency); either a mean corpuscular volume (MCV) of 105 fL or more and platelet count of 120 000/ mcL (to convert to $\times 10^9$/L multiply by 1) or less or an MCV of 105 fL or more and an absolute neutrophil count less than 1200/ mcL (myelodysplasia); a ferritin level less than 40 ng/mL (iron deficiency) (to convert to pmol/L multiply by 2.247); folate levels less than 3.4 ng/mL (folate deficiency) (to convert to nmol/L multiply by 2.266); vitamin B_{12} less than 200 pg/mL (B_{12} deficiency) (to convert to pmol/L multiply by 0.7378); ferritin levels higher than 500 ng/mL and transferrin saturation less than 50% or ferritin levels higher than 40 ng/mL and a history of a medical condition or medication indicating chronic disease or inflammation (anemia of inflammation); haptoglobin levels less than 14 mg/dL (to convert to mg/L multiply by 10) and MCV greater than 100 fL (hemolytic anemia); and IgG, IgA or IgM levels higher than 1.0 g/dL (plasma cell dyscrasia and/or monoclonal gammopathy).

Iron deficiency anemia: Serum ferritin <50 ng/dL, or % transferrin saturation <20% Recommend < 40 ng/mL

Anemia of chronic inflammation: Serum iron <60 ug/dL, saturation >20%, serum ferritin >50 ng/dl without evidence for iron deficiency (Chart information would be helpful. This is reasonable for lab criteria if we don't have any else. We should summarize the CRP values though in this group to confirm ACI represents patients with ACI). Chronic kidney Disease: eGFR <30 mL/min

Myelodysplastic syndromes: MCV >100 fl, platelet count <120 K/uL, or neutrophil count < 1200 K/uL, not attributable to another cause

Vitamin B12 deficiency: B12 levels <200 pg/mL (if low, further confirmation by measurement of methyl malonic acid

Folate deficiency: Low folate levels (serum folate level < 3.4 ng/L)

Hemolytic anemia: Normocytic or macrocytic anemia associated with elevated LDH and low haptoglobin level

Thalassemia trait: MCV <80 fL and red blood cell count within the normal reference range without iron deficiency

Anemia of aging: Does not meet criteria for IDA, ACI, Mixed IDA/ACI, CKD, and MDS

abbvie AndroGel 1.62%

M16-100 – Statistical Analysis Plan (Supplement for Anemia Efficacy Sub-Study)

Version 1.6 – 10 January 2022

Version: 1.6 **Date:** 10-January-2022 **Company ID:** 04122018-00F9F683CEE0E1-00001-

Signed by:	Date:	Meaning of Signature:

February 6, 2022

Shalender Bhasin, MD
Co-Principal Investigator
Brigham and Women's Hospital/Harvard Medical School

Feb 9, 2022

Panagiotis Flevaris, MD, MS
Medical Director
AbbVie Inc.

Date

10 January 2022

Thomas G. Travison, Ph.D.
Biostatistician
Harvard Medical School

February 6, 2022

Karol M. Pencina, Ph.D.
Biostatistician
Brigham and Women's Hospital/Harvard Medical School

Date

February 9, 2022

Xue Li, PhD
Biostatistician
AbbVie Inc.

Date
