# Supplementary material for: Efficacy of Testosterone Replacement Therapy in Correcting Anemia in Men With Hypogonadism: A Randomized Clinical Trial
Source: JAMA Netw Open. 2023 Oct 27;6(10):e2340030. doi: 10.1001/jamanetworkopen.2023.40030 (PMC10611996; doi:10.1001/jamanetworkopen.2023.40030)
Supplement: Supplement 2. — eAppendix. TRAVERSE Study Sites and Committees eFigure 1. Estimated Change From Baseline in Hemoglobin (g/dL) for Participants with Anemia and without Anemia at Baseline eFigure 2. Association of the Standardized Average Change From Baseline in Hemoglobin (g/dL) With the Standardized Average Change in HIS-Q Energy Score in Men With Anemia at Baseline Who Were Assigned to the Active (TRT) Study Group eFigure 3. Prespecified Subgroup Analyses of the Risk of Correction of Anemia in Participants Who Had Anemia at Baseline eFigure 4. Prespecified Subgroup Analyses of the Risk of Increase in Hemoglobin >1g/dL in Participants Who Had Anemia at Baseline eFigure 5. Prespecified Subgroup Analyses of the Risk of Incident Anemia in Participants Who Did Not Have Anemia at Baseline eFigure 6. Sensitivity Analyses of the Risk of Correction of Anemia Among Participants Who Had Anemia at Baseline eFigure 7. Sensitivity Analyses of the Risk of Incident Anemia Among Participants Who Did Not Have Anemia at Baseline eTable 1. Change From Baseline in Red Cell Counts and Hematocrit Levels eTable 2. Changes in Red Blood Cell Indices eTable 3. Changes in Total Testosterone, DHT and Estradiol Levels eTable 4. Listing of Investigator-Reported Adverse Events in Men With Anemia and Men Without Anemia [file jamanetwopen-e2340030-s002.pdf]

Supplemental Online Content

Pencina KM, Travison TG, Artz AS, et al. Efficacy of testosterone replacement therapy in correcting anemia in men with hypogonadism. *JAMA Netw Open.* 2023;6(10):e2340030. doi:10.1001/jamanetworkopen.2023.40030

eAppendix. TRAVERSE Study Sites and Committees

eFigure 1. Estimated Change from Baseline in Hemoglobin (g/dL) for Participants with Anemia and without Anemia at Baseline

eFigure 2. Association of the standardized average change from baseline in hemoglobin (g/dl) with the standardized average change in HIS-Q Energy Score in Men with anemia at baseline who were assigned to the active (TRT) study group

eFigure 3. Prespecified Subgroup Analyses of the Risk of Correction of Anemia in Participants who had Anemia at Baseline

eFigure 4. Prespecified Subgroup Analyses of the Risk of Increase in Hemoglobin >1g/dL in Participants who had Anemia at Baseline

eFigure 5. Prespecified Subgroup Analyses of the Risk of Incident Anemia in Participants who did not have Anemia at Baseline

eFigure 6. Sensitivity Analyses of the Risk of Correction of Anemia Among Participants Who Had Anemia at Baseline

eFigure 7. Sensitivity Analyses of the Risk of Incident Anemia Among Participants Who Did Not Have Anemia at Baseline

eTable 1. Change From Baseline in Red Cell Counts and Hematocrit Levels

eTable 2. Changes in Red Blood Cell Indices

eTable 3. Changes in Total Testosterone, DHT and Estradiol Levels

eTable 4. Listing of Investigator-Reported Adverse Events in Men With Anemia and Men Without Anemia

This supplemental material has been provided by the authors to give readers additional information about their work.

© 2023 Pencina KM et al. *JAMA Network Open.*

eAppendix. TRAVERSE Study Sites and Committees

TRAVERSE Site Investigators:

Number of patients enrolled in parentheses

R. Helm, Investigative Clinical Research of Indiana, LLC, Elwood, IN (93); P. Trueba, Future Care Solution LLC, Miami, FL (91); S. Kester, Clinical Research Center of Florida, Pompano Beach, FL (84); S. Ansari, Longwood Research, Huntsville, AL (68); A. Makhlouf, Hillcroft Medical Clinic, Sugar Land, TX (60); V. Hoon, Advanced Biomedical Research of America Inc., Las Vegas, NV (57); S. Aslam, Northwest Houston Heart Center, Tomball, TX (51); K. Bender, DBC Research, Tamarac, FL (50); J. Cardona, Indago Research and Health Center, Inc., Hialeah, FL (47); A. Dinnerstein, Helix Biomedics, LLC, Boynton Beach, FL (47); B. Wieskopf, North Georgia Clinical Research, Woodstock, GA (47); R. Falcone, Amici Clinical Research, LLC - Raritan, Raritan, NJ (46); J. Mandry, West Orange Endocrinology, P.A., Ocoee, FL (46); E. Shapiro, Eclipse Clinical Research, Tucson, AZ (45); M. Darani, Marvel Clinical Research, LLC, Huntington Beach, CA (44); L. Pearlman, Synexus Clinical Research US, Inc. – Tempe, Chicago, IL (44); S. Nasir, Affinity Clinical Research Institute, Oak Brook, IL (43); R. Heller, Bayview Research Group LLC. Valley Village, CA (42); J. Miracle, Synexus Clinical Research US, Inc. – Tempe, Akron, OH (42); M. Digiovanna, DiGiovanna Family Care Center (DFCC), North Massapequa, NY (41); K. Lucas, Diabetes & Endocrinology Consultants, Morehead City, NC (41); N. Andrawis, Manassas Clinical Research Center, Manassas, VA (39); E. Davidson, New Phase Research & Development, LLC, Knoxville, TN (39); M. Doty, Care Research Center Inc., Miami, FL (39); D. Mishkin, InvesClinic Research Center, Fort Lauderdale, FL (38); C. Brinson, Austin Primary Care Physicians, Pflugerville, TX (37); N. Farris, The Research Group of Lexington, LLC, Lexington, KY (37); G. Kipp, Northwest Clinical Research Center, Bellevue, WA (37); A. Krishna, Diabetes and Endocrinology Associates of Stark County, Inc., Canton, OH (37); P. Moore, ClinRx Research, LLC, Plano, TX (37); G. Yeoman, Founders Research Corporation, Philadelphia, PA (37); M. Allaw, Clinical Research Advantage, Inc. Tempe, Evansville, IN (36); J. Bashour, Synexus – Dallas, Dallas, TX (35); R. Montgomery, Triad Clinical Trials, Greensboro, NC (35); A. Tan, West Coast Research, LLC, Dublin, CA (35); A. Ahmad, East Texas Cardiology, PA, Houston, TX (34); D. Levinson, Chicago Clinical Research Institute, Inc., Chicago, IL (34); G. Shockey, Desert Clinical Research, LLC, Mesa, AZ (34); K. Ayesu, Omega Research Consultants LLC, Orlando, FL (33); L. McGill, Clinical Neuroscience Solutions, Inc. (CNS Healthcare of Memphis), Memphis, TN (33); N. Secrist, Alliance for Multispecialty Research, LLC – Wichita, Wichita, KS (33); N. Gabra, Burke Internal Medicine & Research, Burke, VA (32); J. Kopp, Synexus Clinical Research US, Inc. – Tempe, Anderson, SC (32); L. McGill, Synexus Clinical Research US, Inc. / McGill Family Practice, Omaha, NE (32); N. Morrar, G & L Research, LLC, Foley, AL (32); R. Powell, Velocity Clinical Research - Cincinnati, Cincinnati, OH (32); R. Severance, East Valley Family Physicians, Chandler, AZ (32); P. Bravo, Bravo Health Care Center, North Bay Village, FL (31); R. Broughton, CB Flock Research Corporation, Mobile, AL (31); K. Cruz, Advanced Pharma CR, LLC, Miami, FL (31); S. Gorrela, Spring Family Practice Associates, PA, Spring, TX (30); B. MacGillivray, Discovery Clinical Trials, San Antonio, TX (30); J. Loy, South Florida Wellness & Clinical Research Institute, Margate, FL (29); C. Lunn, Lynn Health Science Institute East, Oklahoma City, OK (29); T. Gilford, Associates in Medicine, PA, Houston, TX (27); P. Grossman, Masters of Clinical Research, Inc., Augusta, GA (27); T. Howard, Medical Affiliated Research Center, Huntsville, AL (27); A. Mendelson, Health Awareness, Inc. – Jupiter, Jupiter, FL (27); E. Riffer, Central Phoenix Medical Clinic, LLC, Phoenix, AZ (27); C. Calinescu, Synexus - Henderson DRS, Henderson, NV (26); P. Levins, Tatum Highlands Medical Associates, Glendale, AZ (26); R. Strzinek, Protenium Clinical Research, Hurst, TX (26); F. Velazquez, Pioneer Research Solutions, Inc., Houston, TX (26); M. Billings, Synexus – Greer, Greer, SC (25); G. Debs-Perez, Harmony Clinical Research, Inc., North Miami Beach, FL (25); K. Lee, Randolph Medical Associates, Asheboro, NC (25); I. Marar, West Broadway Clinic, Council Bluffs, IA (25); R. Toma, Simon-Williamson Clinic, Birmingham, AL (25); A. Adams, AB Clinical Trials, Las Vegas, NV (24); H. Audish, Encompass Clinical Research, Spring Valley, CA (24); M. Azizad, Valley Clinical Trials, Inc., Northridge, CA (24); C. Greiwe, Synexus Clinical Research US, Inc. – Columbus, OH (24); A.L. Lewy-Alterbaum, ALL Medical Research, LLC, Cooper City, FL (24); L. Akright, Northeast Endocrinology Associates, PA, Live Oak, TX (23); A. Al-Karadsheh, The Endocrine Center, Houston, TX (23); J. DeGrauw, Wasatch Peak Family Practice, Layton, UT (23); S. Dua, Valley Renal Medical Group, Northridge, CA (23); W. Jennings, Synexus Clinical Research US, Inc. - San Antonio, San Antonio, TX (23); L. Odekirk, Lynn Institute of Denver, Aurora, CO (23); M. Trevino, Innovative Research, Clearwater, FL (23); J. Venereo, Pharmax Research Clinic, Miami, FL (23); M. Agha, OnSite Clinical Solutions, LLC – Charlotte, Charlotte, NC (22); S. Benjamin, Universal Research Group, LLC, Tacoma, WA (22); M. Efros, Accumed Research Associates, Garden City, NY (22); J.C. Garza, Victorium Clinical

Research, San Antonio, TX (22); V. Gold, FMC Science, LLC, Lampasas, TX (22); J. Kay, Clinical Research Advantage, Inc. - Council Bluffs, Omaha, NE (22); L. Rudolph, New Mexico Clinical Research & Osteoporosis Center, Inc., Albuquerque, NM (22); A. Fatakia, Tandem Clinical Research, LLC, Marrero, LA (21); M. Goisse, Frontier Clinical Research, LLC – Smithfield, Smithfield, PA (21); A. Hartman, Virginia Research Center, LLC, Midlothian, VA (21); K. Vora, Research Integrity, LLC, Owensboro, KY (21); A. Bhargava, Iowa Diabetes and Endocrinology Center (IDEC), West Des Moines, IA (20); L. Murray, Synexus Clinical Research US, Inc. - Pinellas Park, Pinellas Park, FL (20); T. Pluto, Frontier Clinical Research, LLC – Scottdale, Scottdale, PA (20); V. Fragoso, Texas Center for Drug Development, Inc., Houston, TX (19); S. Geller, Centennial Medical Group, Elkridge, MD (19); M. Lopez, Charlotte Heart & Vascular Institute, Port Charlotte, FL (19); R. Orr, Phoenix Medical Group, PC, Peoria, AZ (19); W. Patton, Quality of Life Medical & Research Center, Tuscon, AZ (19); B. Seidman, Seidman Clinical Trials, Delray Beach, FL (19); L. Thurman, IPS Research Company, Oklahoma City, OK (19); H. Tran, Texas Diabetes and Endocrinology - South Austin, Round Rock, TX (19); M. Winnie, Crossroads Clinical Research, LLC, Corpus Christi, TX (19); D. Allison, Hillcrest Clinics, Waco, TX (18); P. Nugent, Synexus Clinical Research US, Inc. – Cincinnati, Cincinnati, OH (18); L. Nunez, New Horizon Research Center, Miami, FL (18); M. Nunez, Synexus – Queens, Jamaica, NY (18); G. Quesada, Veritas Research, Corp., Miami Gardens, FL (18); J. Solis, Centex Studies, Inc. – McAllen, McAllen, TX (18); N. Wick, Meridien Research Inc. – Tampa, Tampa, FL (18); J. Andersen, Meridien Research, Lakeland, FL (17); W. Bowman, Sensible Healthcare, Ocoee, FL (17); D. Butuk, Solaris Clinical Research, Meridian, ID (17); L. Connery, Intend Research, Norman, OK (17); G. Freeman, Health Research of Hampton Roads, Inc. (HRHR), Newport News, VA (17); M. Janik, Accellacare US Inc. of Wilmington, Wilmington, NC (17); R. Klein, Newport Native MD, Inc., Newport Beach, CA (17); R. Lending, Synexus Clinical Research US, Inc. – Tucson, Tucson, AZ (17); T. Lenzmeier, Lenzmeier Family Medicine, Glendale, AZ (17); J. Sandberg, Oakland Medical Research – Michigan, Troy, MI (17); P. Wylie, Preferred Research Partners, Little Rock, AR (17); N. Azad, Edward Hines Jr. VA Hospital, Hines, IL (16); A. Barber, OnSite Clinical Solutions, LLC – Charlotte; Hickory, NC (16); S. Bauer, OnSite Clinical Solutions, LLC – Charlotte, Charlotte, NC (16); H. Maheshwari, Midwest Endocrinology, Crystal Lake, IL (16); F. Munoz, Union Square Medical Associates, PC, Elizabeth, NJ (16); K. Soe, Veterans Affairs North Texas Health Care System (VANTHCS), Dallas, TX (16); E. Soroka, Eugene Soroka MD, Inc., Port Hueneme, CA (16); H. Upadhyay, Sandhill Research, LLC, St. Petersburg, FL (16); J. Agaiby, Clinical Investigation Specialists, Inc., Gurnee, IL (15); C. Bird, Colorado Springs Family Practice, Colorado Springs, CO (15); G. Disick, Imagine Research of Palm Beach County, Boynton Beach, FL (15); L. Duke, DMI Research (Decision Management International Health Care Group, Inc.), Pinellas Park, FL (15); T. Gaskin, Century Clinical Research, Inc., Daytona Beach, FL (15); N. Godbole, Summit Medical Group – Glendale, Glendale, AZ (15); R. Huling, Olive Branch Family Medical Center, Olive Branch, MS (15); D. Jack, Lone Peak Family Medicine, Draper, UT (15); W. Knapp, Med Research One, Florissant, MO (15); G. Ledesma, Arlington Family Health Pavilion, Arlington, TX (15); S. Makam, Mid Hudson Medical Research, PLLC, New Windsor, NY (15); S.A. Mujica Trenche, ALAS Science Clinical Research, Henderson, NV (15); J. Schmidt, North State Clinical Research, Lenoir, NC (15); L. Tharenos, Synexus Clinical Research US, Inc. - St. Louis, St. Louis, MO (15); S. Wilson, Ocean State Clinical Research Partners, LLC, Lincoln, RI (15); R. Anderson, VA Nebraska - Western Iowa Health Care System, Omaha, NE (14); K. Barbel-Johnson, Care Partners Clinical Research, Jacksonville, FL (14); P. Barnhill, Vineland Family Medicine, Whiteville, NC (14); J. Bashour, Synexus – Plano, Plano, TX (14); D. Bouda, Heartland Clinical Research, Omaha, NE (14); J. Daniel, Clinical Research Partners, LLC - Family Medicine / Internal Medicine, Richmond, VA (14); L. Feld, Horizon Clinical Research, LLC, Gilbert, AZ (14); T. Fiel, Fiel Family & Sports Medicine, Tempe, AZ (14); S. Grubb, Waterway Primary Care, LLC - Tabor City, Little River, SC (14); M. Hummel, Fountain Hills Family Practice PC, Fountain Hills, AZ (14); S. Koch, Koch Family Medicine, Morton, IL (14); M. Kramer, MB Clinical Research, Boca Raton, FL (14); J. Pouzar, Centex Studies, Inc. – Houston, Houston, TX (14); G. Trullenque, Floridian Research Institute LLC, Miami, FL (14); M. Welch, Consano Clinical Research, Shavano Park, TX (14); E. Armas, Well Pharma Medical Research, Miami, FL (13); J. Chang, Velocity Clinical Research, North Hollywood, North Hollywood, CA (13); R. Jordan, Center for Clinical Trials of Sacramento, Inc., Sacramento, CA (13); S. Leichter, Endocrine Consultants, PC – Columbus, Columbus, GA (13); R. Perry, Panax Clinical Research, Miami Lakes, FL (13); W. Randall, PriMed Clinical Research, Dayton, OH (13); R. Sastre, APF Research, LLC, Miami, FL (13); E. Bolster, Palmetto Clinical Research (PCR), Summerville, SC (12); A. Cohen, The Endocrine Clinic, PC, Memphis, TN (12); C. Griffin, Lynn Health Science Institute (LHSI), Oklahoma City, OK (12); C. Herman, Quantum Clinical Trials, Miami Beach, FL (12); J. Jacqmein, Jacksonville Center for Clinical Research, Jacksonville, FL (12); B. Khan, Atlanta Vascular Research Foundation - Atlanta Clinical Research Centers, Atlanta, GA (12); I. Lieber, Texas Cardiology Associates of Houston, Kingwood, TX (12); C. Mbogua, Discovery MM Services, Inc. - Houston - Broadway St., Houston, TX (12); A. Murcia, DBC Research USA Corporation, Pembroke Pines, FL (12); R. Patel, Endocrine and Psychiatry Center, Houston, TC (12);

© 2023 Pencina KM et al. *JAMA Network Open.*

H. Stamps, Collierville Medical Specialists, Collierville, TN (12); R. Swerdloff, Lundquist Institute for Biomedical Innovation at Harbor - UCLA Medical Center, Torrance, CA (12); J. Whatley, Centex Studies, Inc., Lake Charles, LA (12); M. Adams, Synexus Clinical Research US, Inc. - Salt Lake City, Murray, UT (11); J. Bailen, First Urology, Jeffersonville, IN (11); M. Bidair, San Diego Clinical Trials, La Mesa, CA (11); C. Breton, International Research Associates, LLC, Miami, FL (11); N. Daboul, Advanced Medical Research, LLC – Maumee, Maumee, OH (11); G. Flippo, Alabama Clinical Therapeutics, LLC, Birmingham, AL (11); M. Franco, Memorial Clinical Associates, PA, Houston, TX (11); G. Funk, Fundamental Research, Gulf Shores, AL (11); E. Gaddam, The Loretto Hospital, Chicago, IL (11); T. Hart, Terence T. Hart MD, Tuscumbia, AL (11); D. Headley, David M. Headley, MD, PA, Port Gibson, MS (11); D. James, The University Of Tennessee Health Science Center, Memphis, TN (11); D. Koontz, Palmetto Institute of Clinical Research, Inc., Pelzer, SC (11); D. McNeil, Optimed Research, LTD, Columbus, OH (11); M. Raikhel, Torrance Clinical Research Institute, Inc., Lomita, CA (11); M. Cornett, American Health Network of IN, LLC – Franklin, Franklin, IN (10); A. Daluga, American Health Network - Family Medicine and Specialty Care Services in Greenfield, Greenfield, IN (10); R. D'Anna, Applied Research Center of Arkansas, Little Rock, AR (10); A. Gosmanov, Albany Stratton VA Medical Center, Albany, NY (10); A. Iranmanesh, Salem VA Medical Center, Salem, VA (10); A. Qureshi, Pioneer Research Solutions, Inc., Beaumont, TX (10); H. Reyes, MediSphere Medical Research Center, Evansville, IN (10); S. Rosenberg, The Iowa Clinic, PC, West Des Moines, IA (10); D. Spiller, South Florida Research Solutions, LLC, Hollywood, FL (10); R. Estevez, Clinical Research of South Nevada, Las Vegas, NV (9); B. Green, B.G. Clinical Research Center, LLC, Little Rock, AR (9); E. Hanna, Internal Medicine Associates, Bridgeton, NJ (9); R. Kastelic, Richard M. Kastelic MD & Associates, PC, Johnstown, PA (9); M. Lawrence, Carteret Medical Group - Morehead City, Morehead City, NC (9); R. Manning, PMG Research of Knoxville, Knoxville, TN (9); K. Maynard, Investigators Research Group, LLC , Indianapolis, IN (9); C. Ng, Tower Urology, Los Angeles, CA (9); L. Phillips, Atlanta VA Medical Center, Decatur, GA (9); S. Plantholt, Maryland Cardiovascular Specialists, Baltimore, MD, (9); R. Powell, Meridien Research Inc. - Spring Hill, Spring Hill, FL (9); O. Ruffin, Trinity Clinical Research, LLC, Tullahoma, TN (9); J. Walsh, Richard L. Roudebush VA Medical Center, Indianapolis, IN (9); M. Woolman, Advanced Research Institute, Ogden, UT (9); S. Arora, Aventiv Research – Columbus, Columbus, OH (8); V. Awasty, Awasty Research Network, LLC, Marion, OH (8); T. Barker, PMG Research of Piedmont Healthcare, Mooresville, NC (8); S. Butman, Verde Valley Medical Center, Tucson, AZ (8); D. Cahn, Foothills Urology, Lakewood, CO (8); S. Dhindsa, Saint Louis University School of Medicine, St. Louis, MO (8); A. Doshi, PrimeCare Medical Group, Houston, TX (8); A. Drabick, Medication Management LLC, Raleigh, NC (8); R. Hood, Discover Research, Inc., Beaumont, TX (8); M. Jardula, Desert Oasis Healthcare, Palm Springs, CA (8); J. Kovac, Urology of Indiana, Indianapolis, IN (8); D. McMullen, Discovery MM Services, Inc. - Missouri City, Missouri City, TX (8); W. Pharr, Medication Management LLC, Greensboro, NC (8); S. Phatak, Connecticut Clinical Research, LLC, Waterbury, CT (8); L. Quintero, International Research Associates, LLC – Hialeah, Hialeah, FL (8); A. Becker, Deaconess Clinic Downtown, Evansville, IN (7); B. Christine, Urology Centers of Alabama, P.C., Homewood, AL (7); W. Fitzgibbons, Skyline Medical Center, Elkhorn, NE (7); D. Fitz-Patrick, East-West Medical Research Institute, Honolulu, HI (7); N. Fraser, Troy Internal Medicine, P.C. - Research Division, Troy, MI (7); B. Gilbert, Office of Bruce R. Gilbert, MD, Great Neck, NY (7); I. Goldstein, San Diego Sexual Medicine, San Diego, CA (7); J. Haffizulla, Precision Clinical Research, LLC – Lauderdale Lakes, Lauderdale Lakes, FL (7); J. Lillo, Elite Clinical Studies, LLC, Phoenix, AZ (7); T. Nguyen, Solutions Through Advanced Research, Inc., Jacksonville, FL (7); E. Riley, Self Medical Group - Advanced Research Associates, Hodges, SC (7); D. Storey, American Health Network of IN, LLC – Avon, Avon, IN (7); S. Tebi, Care Access Research, Santa Clarita, CA (7); D. Uba, Rapha Institute for Clinical Research, Fayetteville, NC (7); J. Wayne, Clinical Trials Research, Lincoln, CA (7); D. Ajani, Discovery MM Services, Inc.– Katy, Houston, TX (6); E. Christofides, Endocrinology Research Associates, Columbus, OH (6); D. Francyk, Family Practice Specialists, Phoenix, AZ (6); D. Huffman, University Diabetes & Endocrine Consultants, Chattanooga, TN (6); S. Malempati, Tampa Bay Medical Research, Clearwater, FL (6); A. Odugbesan, Physicians Research Associates, LLC, Lawrenceville, GA (6); B. Pierpont, Advance Medical Research, Inc., St. Petersburg, FL (6); M. Rausch, Heartland Research Associates, LLC, El Dorado, KS (6); N. Razzaque, Synexus Clinical Research US, Inc. - West Florissant Internists, Bridgeton, MO (6); K. Rictor, SFM Clinical Trials, Scotland, PA (6); J. Stewart, Family Medicine Associates of Texas, PA, Carrollton, TX (6); D. Tripathy, South Texas Veterans Health Care System - Audie L. Murphy VA Hospital, San Antonio, TX (6); T. Briskin, Velocity Clinical Research - Cleveland, Cleveland, OH (5); K. Cohen, New West Physicians, PC, Golden, CO (5); A. Dang, Facey Medical Group, Mission Hills, CA (5); K. Fox, Family Medical Associates of Highland Park, LLC, Levittown, PA (5); S. Freedman, Sheldon J. Freedman, MD, Ltd., Las Vegas, NV (5); A. George, Seven Corners Medical Center, Falls Church, VA (5); E. Goldfischer, Premier Medical Group - Urology Division, Poughkeepsie, NY (5); R. Hollister, Lynn Institute of the Rockies, Colorado Springs, CO (5); R. Jacks, Hill Country Medical Associates, New Braunfels, TX (5); K. Kelley, Biofortis Clinical Research, Inc.,

© 2023 Pencina KM et al. *JAMA Network Open.*

Addison, IL (5); R. Mills, PMG Research of Charleston, Mt Pleasant, SC (5); R. Mohseni, Catalina Research Institute, LLC, Montclair, CA (5); F. Saba, Professional Health Care of Pinellas, Inc., St. Petersburg, FL (5); J. Sensenbrenner, PMG Research of Charlotte, Charlotte, NC (5); E. Sorial, Prima CARE, P.C., Fall River, MA (5); P. Wakefield, PMG Research of Knoxville, Knoxville, TN (5); L. Whitlock, Primary Care Group, LLC, Memphis, TN (5); M. Ampajwala, ACRC Trials, Frisco, TX (4); J. Borders, Drs. Borders, Hood, and Associates, Lexington, KY (4); P. Bradley, Meridian Clinical Research, LLC – Savannah, Savannah, GA (4); O.G. Brkic-Vukotic, Atlanta Center for Clinical Research, Roswell, GA (4); A. Brockmyre, Holston Medical Group, Bristol, TN (4); J. Chehade, University of Florida Health Endocrinology – Emerson, Jacksonville, FL (4); R. Harris, DeGarmo Institute of Medical Research, Greer, SC (4); N. Jaffrani, Alexandria Cardiology Clinic, Alexandria, LA (4); J. Kaminetsky, Manhattan Medical Research, New York, NY (4); G. Ledger, Mercy Clinic Endocrinology, LLC, Springfield, MO (4); S. Lerman, The Center for Diabetes and Endocrine Care – Hollywood, Ft. Lauderdale, FL (4); A. Mabaquiao, TriWest Research Associates, El Cajon, CA (4); I.J. Madu, Diabetes Associates Medical Group, Orange, CA (4); R. Myers, "Clinic-LJ" Ltd, Ponte Vedra, FL (4); S. Nakhle, Palm Research Center, Inc., Las Vegas, NV(4); J. Perez, South Texas Cardiovascular Consultants, San Antonio, TX (4); W. Rust, Heritage Valley Medical Group, Beaver, PA (4); J. Serje, NY Total Medical Care, P.C., Brooklyn, NY (4); L. Smith, WVVA HealthCare Alliance, PC - Valley Medical Associates Inc., Lewisburg, WV (4); M. Tellez, Pacific Oaks Medical Group, Beverly Hills, CA (4); L. Belkoff, MidLantic Urology - Bala Cynwyd, Bala Cynwyd, PA (3); L. Berman, OnSite Clinical Solutions, LLC – Charlotte, Charlotte, NC (3); J. Cochran, Urology Clinics of North Texas, Dallas, TX (3); C. Cone, Montana Medical Research, Missoula, MT (3); K. Ellis, Clinical Research Associates of Tidewater, Norfolk, VA (3); S.M. Harman, Phoenix VA Health Care System, Phoenix, AZ (3); L. Herman, Herman Clinical Research, LLC, Suwanee, GA (3); J. Kirby, PMG Research of Knoxville, Jefferson City, TN (3); D. Liljenquist, Rocky Mountain Diabetes and Osteoporosis Center, Idaho Falls, ID (3); E. Morawski, Holston Medical Group, Kingsport, TN (3); N.C. Morcos, Syrentis Clinical Research, Santa Ana, CA (3); M. Patel, LaPorte County Institute for Clinical Research, Inc., Michigan City, IN (3); W. Penny, VA San Diego Healthcare System, San Diego, CA (3); E. Portnoy, Millennium Clinical Trials, Thousand Oaks, CA (3); M. Shanik, Endocrine Associates of Long Island, P.C., Smithtown, NY (3); H. Bagga, AGH Internal Medicine – Northside, Pittsburgh, PA (2); V. Bland, Bland Clinic, PA, Greensboro, NC (2); P. Bressler, North Texas Endocrine Center, Dallas, TX (2); J. Condit, American Health Network of Indiana – Muncie, Muncie, IN (2); A. Latorre, Applemed Research, Inc., Miami, FL (2); R. Leon, IMIC Inc., Palmetto Bay, FL (2); M. Marcelli, Michael E. DeBakey VA Medical Center, Houston, TX (2); M. Palatnik; Allied Clinical Research, LLC, Gold River; CA (2); B. Rizzardi, Velocity Clinical Research, Salt Lake City, West Jordan, UT (2); M. Vaughn, Cahaba Research, Inc., Birmingham, AL (2); J. Vazquez-Tanus, Research And Cardiovascular Corp., Ponce, PR (2); J. Willis, San Gabriel Clinical Research, Georgetown, TX (2); A. Alcantara, Centro de Endocrinologia Alcantara-Gonzalez, Bayamon, PR (1); K. Blaze, South Broward Research, LLC, Pembroke Pines, FL (1); W. Clark, Alaska Urological Institute, Anchorage, AK (1); G. Cortes-Maisonet, GCM Medical Group, PSC., San Juan, PR (1); T. Dixon, Tanner Clinic – Clinton, Clinton, UT (1); P. Greenberg, James J. Peters VA Medical Center, Bronx, NY (1); A. Gupta, Dayton VA Medical Center, Dayton , OH (1); C. Harper, Meridian Clinical Research, LLC – Norfolk, Norfolk, NE (1); H. Kerr, Cincinnati Veterans Affairs Medical Center, Cincinnati, OH (1); R. Leggett, Crossroads Clinical Research, LLC, Victoria, TX (1); E. Levin, VA Long Beach Healthcare System, Long Beach, CA (1); V. Mahabadi, Olive View - UCLA Medical Center, Sylmar, CA (1); B. Miranda, University of Miami - Miller School of Medicine, Miami, FL (1); J.H. Peniston, Thomas Jefferson University Hospital, Feasterville-Trevose, PA (1); A. Seftel, Cooper University Health Care, Camden, NJ (1); N. Sunkara, Optimum Clinical Research, Optimum Clinical Research, UT (1); A. Wokhlu, Malcom Randall VA Medical Center, Gainesville, FL (1); B. Wolf, Allied Clinical Research – Reno, Reno, NV (1); D. Wynn, Wright Clinical Research, Alabaster, AL (1); The following sites were also initiated to begin study activities: R. Adler, Hunter Holmes McGuire VA Medical Center, Richmond, VA; A. Ahmad, Cardiovascular Association, PLLC, Humble, TX; O. Barnum, KAMP Medical Research, Inc., Natchitoches, LA; A. Clark, VA Pittsburgh Healthcare System, Pittsburgh, PA; D. Cohen-Neamie, Charter Research LLC, Winter Park, FL; A. Comulada Rivera, Advanced Clinical Research – Bayamon, Bayamon, PR; B. Cowan, Urology Associates of Colorado, Englewood, CO; M. Dawson, ACRC Trials, Austin, TX; A. Dobs, Johns Hopkins University School of Medicine, Baltimore, MD; J. Elsen, Pharmakon Inc., Evergreen Park, IL; J. Elsen, DuPage Medical Group - Downers Grove - 31st Street, Evergreen Park, IL; B. Essink, Meridian Clinical Research, LLC – Omaha, Omaha, NE; R. Farsad, Diagnamics, Encinitas, CA; B. First, Ritchken & First MDs, San Diego, CA; B. Frandsen, Sound Medical Research, Port Orchard, WA; A. Frisoli, Amici Clinical Research, LLC – Hoboken, Hoboken, NJ; N. Gabrail, Clinical Research Trials, LLC, Canton, OH; R. Garcia, Covenant Clinical Research, PA, San Antonio, TX; S. Georgeson, Medicor Cardiology, Bridgewater, NJ; J. Gilbert, St. Jude Hospital Yorba Linda, Fullerton, CA; L. Hernandez-Vazquez, Emanuelli Research and Development Center, Arecibo, PR; A. Hoffman, VA Palo Alto Health Care System, Palo Alto, CA; R. Hunter, ARA Arizona Research Associates, Tucson, AZ; D. Hurley, Family First

Medical Care, LLC, Goose Creek, SC; L. Jenkins, The Ohio State University Wexner Medical Center - Department of Urology, Columbus, OH; A. Kabour, ID Clinical Research, Ltd., Toledo, OH; D. Kayne, The Medical Group of Encino, Encino, CA; Y. Kidokoro, Integrated Research Center, Inc., San Diego, CA; D. Lee, Irvine Center for Clinical Research, Irvine, CA; A. Matsumoto, VA Puget Sound Health Care System, Seattle, WA; A. Mills, Anthony Mills, MD, Inc., Los Angeles, CA; D. Mobley, Methodist Urology Associates, Houston, TX; S.P. Mokshagundam, University of Louisville, Louisville, KY; M. Pahor, University of Florida College of Medicine, Gainesville, FL; B. Purushottam, Monument Health Clinical Research, Rapid City, SD; O. Raheem, Tulane Medical Center, New Orleans, LA; S. Randhawa, S & W Clinical Research, Fort Lauderdale, FL; S. Shah, St. Joseph's Medical Associates, Inc., Stockton, CA; M. Syed, Endocrinology Associates of Armstrong – Indiana, Indiana, PA; S. Thomson, Veterans Affairs (VA) Health Care System – Tucson, Tucson, AZ; L. Torres, DeLeon Research, PLLC, Plano, TX

TRAVERSE Anemia Study Committee

The Diabetes Study Committee, led by the Research Program in Men's Health, Aging and Metabolism at the Brigham and Women's Hospital, Harvard Medical School IN Boston, MA, USA, designed the study, provided operational oversight to its implementation, and crafted its statistical analysis plan.

Chair: Shalender Bhasin, MB, BS; Members: Andrew Artz, MD; Karol M. Pencina, PhD. (Anemia Study Statistician), Thomas G. Travison, PhD (Anemia Study Statistician); Thiago Gugliano-Juca, MD; PhD; Lauren Wilson, RNP; Kathleen Wannemuehler, Ph.D. (Anemia Study Statistician).

AbbVie Staff: ***Panagiotis Flevaris, MD, PhD; Xue Li, PhD; Anna Chan, PharmD; Elena Dubcenco, MD, MS; Sandra Fukumoto, MBA; Rachel Preuss, BS; Samantha Phillips;*** Thomas J. Korellis, BS.

Funding

The trial was funded by a consortium of testosterone manufacturers led by AbbVie, Inc. (North Chicago, IL) with additional financial support provided by Endo Pharmaceuticals (Malvern, PA), Acerus Pharmaceuticals Corporation (Ontario, Canada), and Upsher-Smith Laboratories, LLC (Maple Grove, MN).

Data and Safety Monitoring Committee

John H. Alexander, MD, MHSc (Chairman), Duke Clinical Research Institute, Duke University, Durham, NC; William Bremner, MD, PhD, University of Washington, Seattle, WA; Eric Klein, MD, Cleveland Clinic, Cleveland, OH; Darren K. McGuire, MD MHSc, University of Texas Southwestern Medical Center, Dallas, TX; Janet Wittes, PhD, Wittes LLC, Washington, D.C.; Renato D. Lopes, MD, PhD (Observer, non-voting), Duke Clinical Research Institute, Durham, NC; Andrew Armstrong, MD, ScM (ad hoc consult), Duke University Medical Center, Duke Cancer Institute Center for Prostate and Urologic Cancers, Durham, NC

Independent Statistician for the Data Monitoring Committee

Kevin A. Buhr, PhD (non-voting Reporting Statistician), University of Wisconsin Statistical Data Analysis Center, Madison, WI

© 2023 Pencina KM et al. *JAMA Network Open.*

eFigure 1. Estimated Change from Baseline in Hemoglobin (g/dL) for Participants with Anemia (Panel A) and without Anemia (panel B) at Baseline.

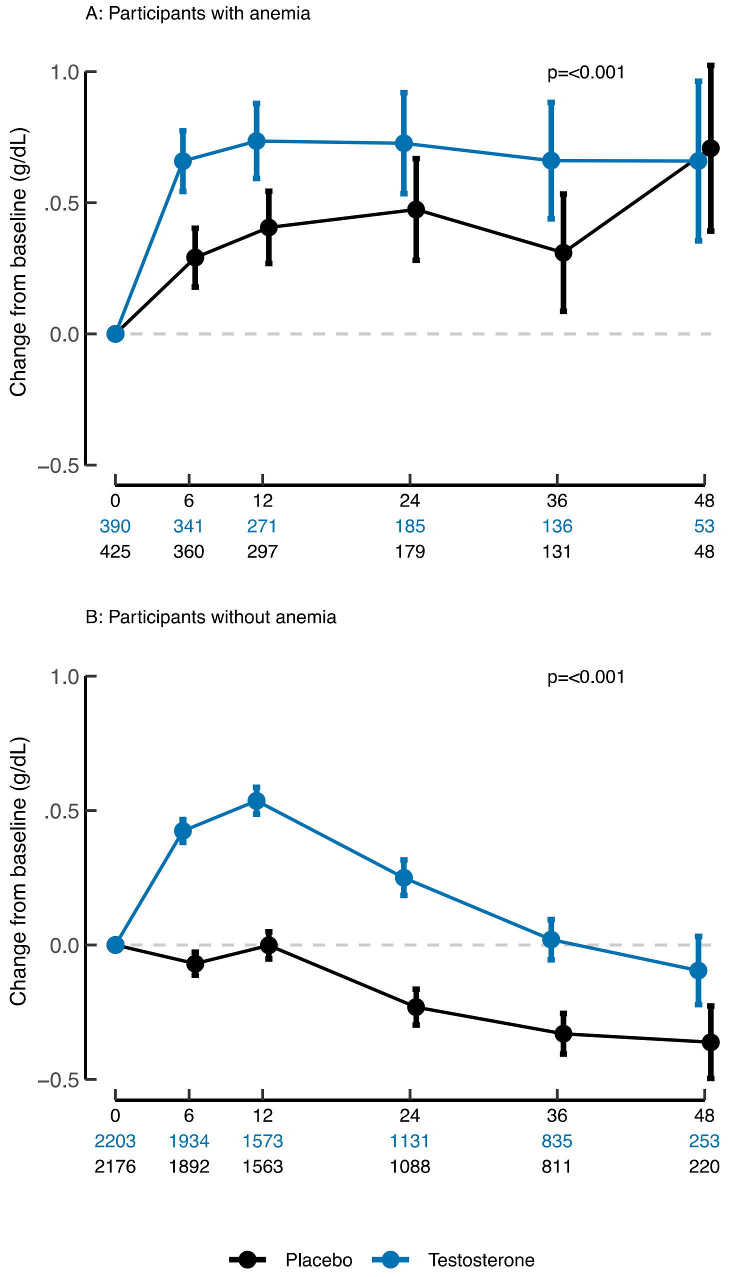

Legend: A linear mixed model was fit with fixed effects for treatment, visit, and treatment-visit interaction, baseline value and pre-existing cardiovascular disease and a random per-subject repeated measures effect with an unstructured covariance matrix.

The LS Means and the corresponding 95% confidence intervals are shown by treatment group and time point. The omnibus test p values were derived separately for participants with anemia and without anemia from a test of the null hypothesis of no difference between the TRT and placebo groups across all time points.

© 2023 Pencina KM et al. *JAMA Network Open.*

eFigure 2. Association of the standardized average change from baseline in hemoglobin (g/dl) with the standardized average change in HIS-Q Energy Score in Men with anemia at baseline who were assigned to the active (TRT) study group.

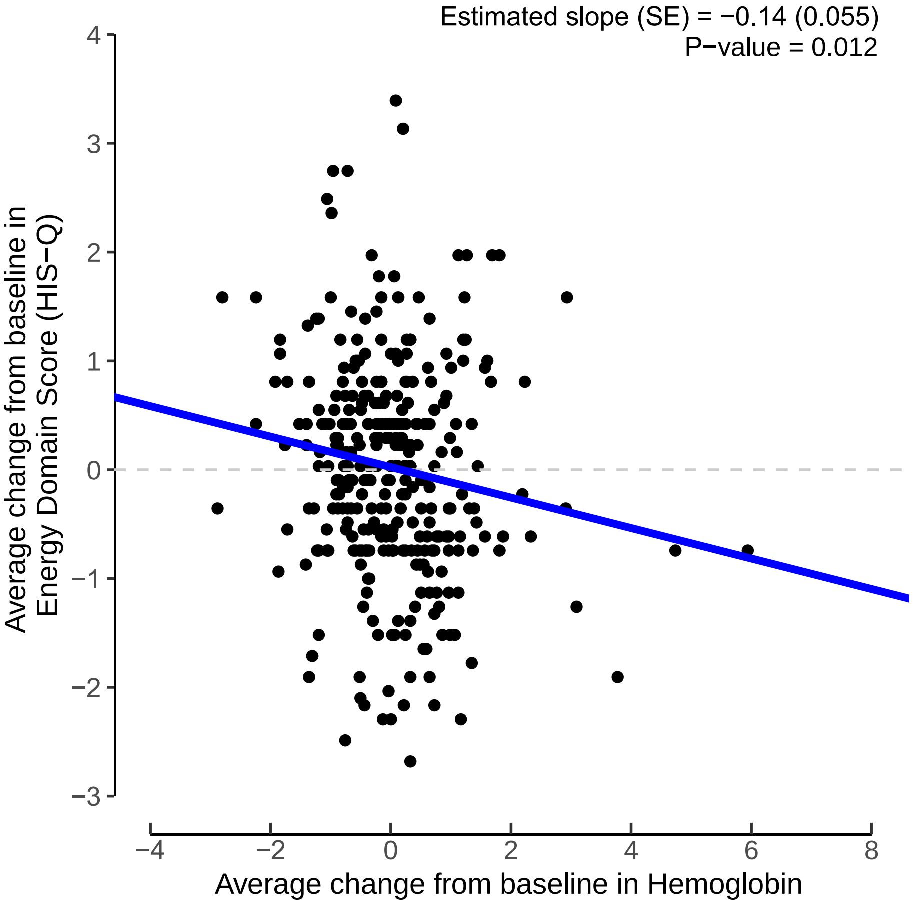

Legend. Association of standardized average change of hemoglobin level with standardized average change in HIS-Q energy domain score in men with anemia at baseline who were assigned to the active treatment group (TRT). The points represent each participant's standardized average change from baseline across visits 6, 12, and 24 months. Shown are the estimated slope and standard deviation (SD) and p-value testing the null hypothesis: slope = 0 derived from a linear model adjusting for pre-existing cardiovascular disease,

© 2023 Pencina KM et al. *JAMA Network Open.*

eFigure 3. Prespecified Subgroup Analyses of the Risk of Correction of Anemia in Participants who had Anemia at Baseline.

A: Prior CVD

Month	TRT	Placebo	RR (95% CI)
	Number (%) Participants Experiencing Events		
6	70/204 (34%)	56/226 (25%)	1.38 (1.03−1.86)
12	79/195 (41%)	67/214 (31%)	1.28 (0.99−1.67)
24	63/156 (40%)	57/169 (34%)	1.16 (0.87−1.55)
36	39/ 97 (40%)	37/106 (35%)	1.09 (0.78−1.52)
48	14/ 31 (45%)	15/ 31 (48%)	0.89 (0.56−1.42)

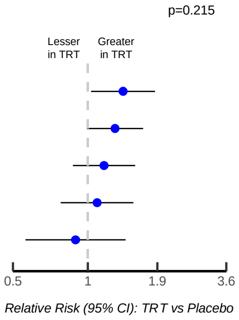

B: No Prior CVD

Month	TRT	Placebo	RR (95% CI)
	Number (%) Participants Experiencing Events		
6	73/145 (50%)	47/149 (32%)	1.60 (1.20−2.13)
12	73/143 (51%)	55/146 (38%)	1.37 (1.05−1.78)
24	61/134 (46%)	38/138 (28%)	1.66 (1.20−2.30)
36	55/119 (46%)	39/123 (32%)	1.48 (1.08−2.03)
48	27/ 61 (44%)	23/ 66 (35%)	1.23 (0.83−1.81)

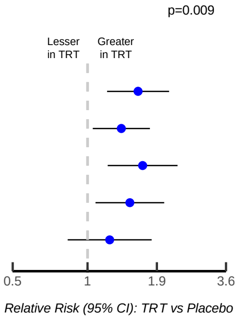

C: >= 65 years

Month	TRT	Placebo	RR (95% CI)
	Number (%) Participants Experiencing Events		
6	77/187 (41%)	46/207 (22%)	1.86 (1.37−2.52)
12	79/181 (44%)	59/199 (30%)	1.46 (1.12−1.92)
24	61/155 (39%)	44/168 (26%)	1.56 (1.13−2.16)
36	45/115 (39%)	35/124 (28%)	1.43 (1.01−2.05)
48	22/ 45 (49%)	13/ 43 (30%)	1.29 (0.79−2.12)

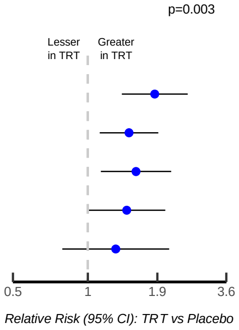

D: <65 years

Month	TRT	Placebo	RR (95% CI)
	Number (%) Participants Experiencing Events		
6	66/162 (41%)	57/168 (34%)	1.21 (0.91−1.60)
12	73/157 (46%)	63/161 (39%)	1.20 (0.93−1.54)
24	63/135 (47%)	51/139 (37%)	1.22 (0.92−1.62)
36	49/101 (49%)	41/105 (39%)	1.15 (0.85−1.55)
48	19/ 47 (40%)	25/ 54 (46%)	0.93 (0.63−1.38)

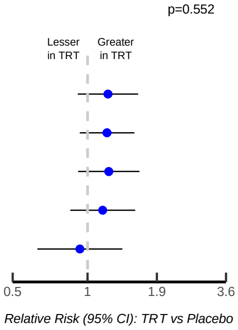

E: Testosterone <250

Month	TRT	Placebo	RR (95% CI)
	Number (%) Participants Experiencing Events		
6	107/258 (41%)	79/281 (28%)	1.49 (1.18−1.88)
12	110/249 (44%)	89/270 (33%)	1.33 (1.07−1.66)
24	88/215 (41%)	79/235 (34%)	1.21 (0.95−1.54)
36	70/162 (43%)	59/175 (34%)	1.25 (0.97−1.63)
48	30/ 73 (41%)	31/ 76 (41%)	0.96 (0.67−1.37)

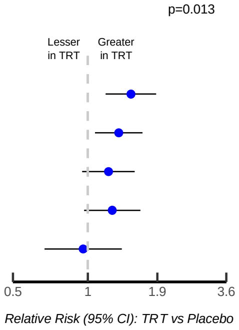

F: Testosterone >= 250

Month	TRT	Placebo	RR (95% CI)
	Number (%) Participants Experiencing Events		
6	36/ 91 (40%)	24/ 94 (26%)	1.53 (0.99−2.33)
12	42/ 89 (47%)	33/ 90 (37%)	1.31 (0.93−1.84)
24	36/ 75 (48%)	16/ 72 (22%)	2.15 (1.32−3.50)
36	24/ 54 (44%)	17/ 54 (31%)	1.39 (0.84−2.30)
48	11/ 19 (58%)	7/ 21 (33%)	1.66 (0.93−2.98)

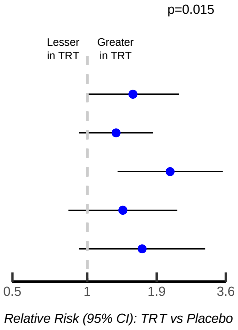

G: Black

Month	TRT	Placebo	RR (95% CI)
	Number (%) Participants Experiencing Events		
6	42/108 (39%)	29/110 (26%)	1.43 (0.97−2.12)
12	52/108 (48%)	34/107 (32%)	1.46 (1.04−2.05)
24	42/ 98 (43%)	27/ 96 (28%)	1.47 (0.99−2.18)
36	31/ 75 (41%)	25/ 78 (32%)	1.28 (0.85−1.93)
48	14/ 34 (41%)	16/ 38 (42%)	0.98 (0.58−1.64)

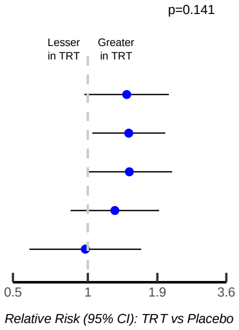

H: White

Month	TRT	Placebo	RR (95% CI)
	Number (%) Participants Experiencing Events		
6	98/229 (43%)	73/257 (28%)	1.53 (1.20−1.95)
12	96/218 (44%)	87/245 (36%)	1.25 (1.01−1.57)
24	76/182 (42%)	67/204 (33%)	1.28 (0.98−1.66)
36	60/134 (45%)	49/144 (34%)	1.28 (0.96−1.71)
48	23/ 53 (43%)	22/ 56 (39%)	1.05 (0.70−1.57)

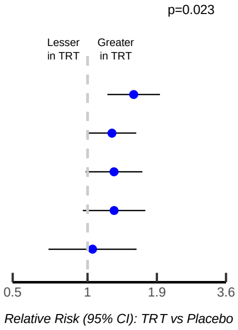

© 2023 Pencina KM et al. *JAMA Network Open.*

Legend. Frequencies and relative risks of correction of anemia with the corresponding 95% confidence intervals for prespecified subgroups (age <65, 65 or older; pre-existing CVD or not; baseline testosterone <250 or ≥250; racial group White or Black) of study participants who had anemia at baseline are shown by treatment group and time point from four separate 3-way interaction models. The contrast test p value for the difference between treatment groups across all time points within a subpopulation is shown. The p values for the contrast test of interaction that tested if there is a difference in treatment effect between subpopulations across all time points were as follows: Prior CVD vs no prior CVD, p = 0.628; baseline testosterone <250 versus ≥ 250 ng/dL, p = 0.051; age <65 versus ≥65 years, p = 0.456; and race Black versus White, p = 0.902. A compound symmetric covariance matrix was assumed in all models.

© 2023 Pencina KM et al. *JAMA Network Open.*

eFigure 4. Prespecified Subgroup Analyses of the Risk of Increase in Hemoglobin > 1g/dL in Participants who had Anemia at Baseline.

A: Prior CVD

	Number (%) Participants Experiencing Events		
Month	TRT	Placebo	RR (95% CI)
6	60/204 (29%)	39/226 (17%)	1.70 (1.19−2.43)
12	56/195 (29%)	52/214 (24%)	1.19 (0.86−1.65)
24	45/156 (29%)	44/169 (26%)	1.09 (0.76−1.56)
36	25/ 97 (26%)	26/106 (25%)	0.96 (0.60−1.53)
48	8/ 31 (26%)	14/ 31 (45%)	0.46 (0.22−0.95)

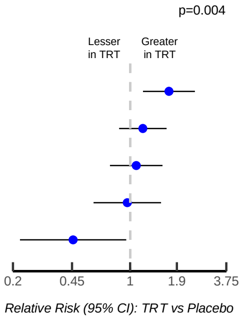

B: No Prior CVD

	Number (%) Participants Experiencing Events		
Month	TRT	Placebo	RR (95% CI)
6	54/145 (37%)	23/149 (15%)	2.41 (1.57−3.71)
12	54/143 (38%)	25/146 (17%)	2.23 (1.47−3.38)
24	45/134 (34%)	24/138 (17%)	1.92 (1.26−2.93)
36	44/119 (37%)	25/123 (20%)	1.74 (1.16−2.61)
48	23/ 61 (38%)	15/ 66 (23%)	1.43 (0.90−2.26)

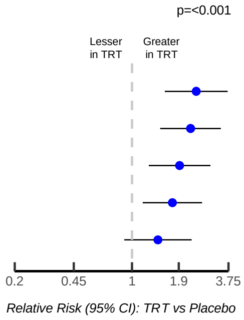

C: >= 65 years

	Number (%) Participants Experiencing Events		
Month	TRT	Placebo	RR (95% CI)
6	64/187 (34%)	30/207 (14%)	2.36 (1.61−3.47)
12	60/181 (33%)	39/199 (20%)	1.71 (1.20−2.42)
24	42/155 (27%)	33/168 (20%)	1.40 (0.94−2.10)
36	32/115 (28%)	27/124 (22%)	1.23 (0.80−1.91)
48	15/ 45 (33%)	12/ 43 (28%)	0.83 (0.46−1.49)

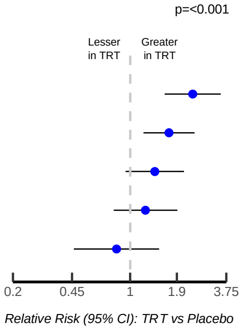

D: <65 years

	Number (%) Participants Experiencing Events		
Month	TRT	Placebo	RR (95% CI)
6	50/162 (31%)	32/168 (19%)	1.62 (1.10−2.39)
12	50/157 (32%)	38/161 (24%)	1.37 (0.95−1.97)
24	48/135 (36%)	35/139 (25%)	1.35 (0.94−1.93)
36	37/101 (37%)	24/105 (23%)	1.35 (0.90−2.01)
48	16/ 47 (34%)	17/ 54 (31%)	0.96 (0.59−1.56)

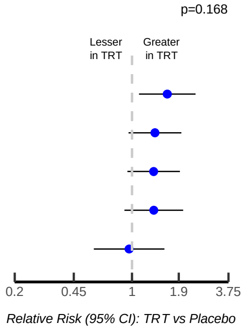

E: Testosterone <250

	Number (%) Participants Experiencing Events		
Month	TRT	Placebo	RR (95% CI)
6	89/258 (34%)	44/281 (16%)	2.21 (1.61−3.04)
12	82/249 (33%)	55/270 (20%)	1.63 (1.21−2.19)
24	63/215 (29%)	54/235 (23%)	1.29 (0.94−1.77)
36	53/162 (33%)	38/175 (22%)	1.37 (0.98−1.93)
48	25/ 73 (34%)	23/ 76 (30%)	0.91 (0.60−1.39)

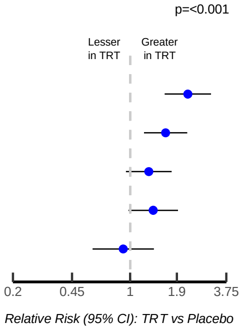

F: Testosterone >= 250

	Number (%) Participants Experiencing Events		
Month	TRT	Placebo	RR (95% CI)
6	25/ 91 (27%)	18/ 94 (19%)	1.43 (0.84−2.44)
12	28/ 89 (31%)	22/ 90 (24%)	1.33 (0.82−2.13)
24	27/ 75 (36%)	14/ 72 (19%)	1.69 (0.99−2.87)
36	16/ 54 (30%)	13/ 54 (24%)	1.11 (0.61−2.03)
48	6/ 19 (32%)	6/ 21 (29%)	0.96 (0.43−2.13)

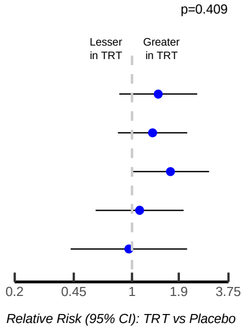

G: Black

	Number (%) Participants Experiencing Events		
Month	TRT	Placebo	RR (95% CI)
6	27/108 (25%)	14/110 (13%)	1.93 (1.07−3.48)
12	30/108 (28%)	24/107 (22%)	1.23 (0.77−1.96)
24	26/ 98 (27%)	20/ 96 (21%)	1.18 (0.71−1.97)
36	21/ 75 (28%)	15/ 78 (19%)	1.25 (0.71−2.21)
48	13/ 34 (38%)	11/ 38 (29%)	0.90 (0.45−1.80)

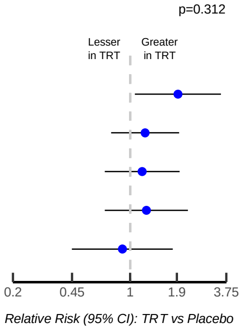

H: White

	Number (%) Participants Experiencing Events		
Month	TRT	Placebo	RR (95% CI)
6	85/229 (37%)	47/257 (18%)	2.06 (1.51−2.80)
12	77/218 (35%)	53/245 (22%)	1.67 (1.24−2.26)
24	60/182 (33%)	48/204 (24%)	1.42 (1.03−1.95)
36	47/134 (35%)	36/144 (25%)	1.30 (0.92−1.84)
48	16/ 53 (30%)	18/ 56 (32%)	0.85 (0.53−1.35)

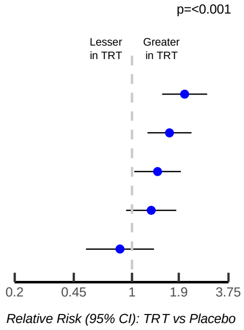

© 2023 Pencina KM et al. *JAMA Network Open.*

Legend. Frequencies and relative risks of increase in hemoglobin >1 g/dL with the corresponding 95% confidence intervals for prespecified subgroups (age <65, 65 or older; pre-existing CVD or not; baseline testosterone <250 or ≥250; racial group White or Black) of study participants who had anemia at baseline are shown by treatment group and time point from four separate 3-way interaction models. The contrast test p value for the difference between treatment groups across all time points within a subpopulation is shown. The p values for the contrast test of interaction that tested if there is a difference in treatment effect between subpopulations across all time points were as follows: Prior CVD vs no prior CVD, p = 0.041; baseline testosterone <250 versus ≥ 250 ng/dL, p =0.235; age <65 versus ≥65 years, p = 0.762 and race Black versus White, p =0.897. A compound symmetric covariance matrix was assumed in all models.

© 2023 Pencina KM et al. *JAMA Network Open.*

eFigure 5. Prespecified Subgroup Analyses of the Risk of Incident Anemia in Participants who did not have Anemia at Baseline.

A: Prior CVD

Month	TRT	Placebo	RR (95% CI)
	Number (%) Participants Experiencing Events		
6	97/1041 (9%)	122/1045 (12%)	0.80 (0.62−1.03)
12	91/993 (9%)	108/997 (11%)	0.83 (0.64−1.08)
24	91/833 (11%)	114/829 (14%)	0.81 (0.63−1.04)
36	54/509 (11%)	68/523 (13%)	0.91 (0.68−1.23)
48	17/162 (10%)	19/149 (13%)	0.80 (0.49−1.30)

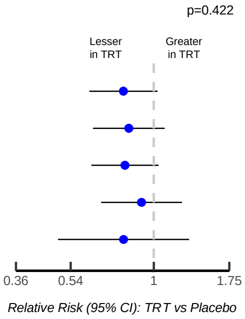

B: No Prior CVD

Month	TRT	Placebo	RR (95% CI)
	Number (%) Participants Experiencing Events		
6	46/956 (5%)	81/913 (9%)	0.54 (0.38−0.77)
12	46/941 (5%)	63/897 (7%)	0.70 (0.49−1.02)
24	83/913 (9%)	93/848 (11%)	0.84 (0.63−1.11)
36	81/838 (10%)	99/777 (13%)	0.77 (0.59−1.02)
48	34/408 (8%)	32/350 (9%)	0.89 (0.62−1.28)

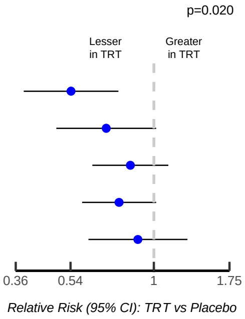

C: >= 65 years

Month	TRT	Placebo	RR (95% CI)
	Number (%) Participants Experiencing Events		
6	66/948 (7%)	100/902 (11%)	0.63 (0.47−0.85)
12	59/919 (6%)	90/872 (10%)	0.62 (0.46−0.85)
24	96/831 (12%)	121/770 (16%)	0.75 (0.58−0.96)
36	70/649 (11%)	94/607 (15%)	0.75 (0.57−0.99)
48	25/265 (9%)	29/213 (14%)	0.67 (0.45−0.99)

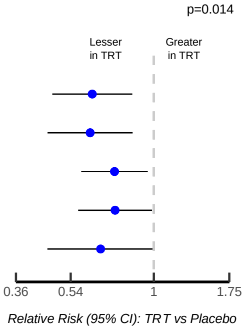

D: <65 years

Month	TRT	Placebo	RR (95% CI)
	Number (%) Participants Experiencing Events		
6	77/1049 (7%)	103/1056 (10%)	0.75 (0.57−0.99)
12	78/1015 (8%)	81/1022 (8%)	0.96 (0.71−1.30)
24	78/915 (9%)	86/907 (9%)	0.93 (0.70−1.23)
36	65/698 (9%)	73/693 (11%)	0.96 (0.72−1.29)
48	26/305 (9%)	22/286 (8%)	1.15 (0.76−1.74)

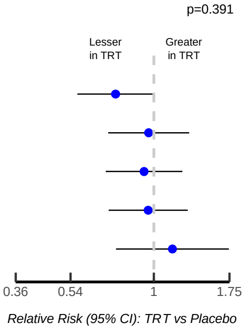

E: Testosterone <250

Month	TRT	Placebo	RR (95% CI)
	Number (%) Participants Experiencing Events		
6	99/1344 (7%)	142/1301 (11%)	0.68 (0.53−0.87)
12	88/1306 (7%)	122/1255 (10%)	0.70 (0.53−0.91)
24	116/1176 (10%)	138/1116 (12%)	0.84 (0.67−1.05)
36	92/907 (10%)	121/862 (14%)	0.81 (0.63−1.03)
48	36/391 (9%)	38/333 (11%)	0.79 (0.56−1.13)

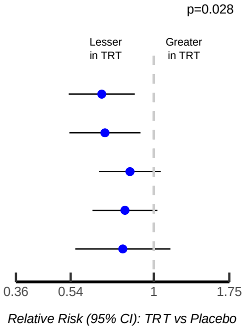

F: Testosterone >= 250

Month	TRT	Placebo	RR (95% CI)
	Number (%) Participants Experiencing Events		
6	44/653 (7%)	61/657 (9%)	0.73 (0.50−1.05)
12	49/628 (8%)	49/639 (8%)	1.00 (0.68−1.46)
24	58/570 (10%)	69/561 (12%)	0.81 (0.59−1.11)
36	43/440 (10%)	46/438 (11%)	0.94 (0.66−1.35)
48	15/179 (8%)	13/166 (8%)	1.09 (0.68−1.76)

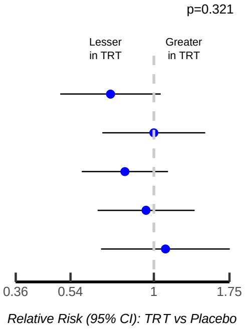

G: Black

Month	TRT	Placebo	RR (95% CI)
	Number (%) Participants Experiencing Events		
6	31/295 (11%)	48/273 (18%)	0.59 (0.39−0.90)
12	36/290 (12%)	35/265 (13%)	0.93 (0.60−1.43)
24	42/260 (16%)	44/237 (19%)	0.87 (0.60−1.27)
36	37/200 (18%)	35/188 (19%)	1.02 (0.68−1.51)
48	11/ 86 (13%)	10/ 80 (12%)	0.81 (0.42−1.55)

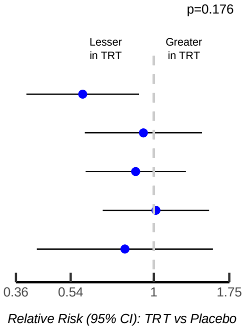

H: White

Month	TRT	Placebo	RR (95% CI)
	Number (%) Participants Experiencing Events		
6	107/1641 (7%)	148/1618 (9%)	0.72 (0.57−0.91)
12	97/1585 (6%)	133/1565 (8%)	0.72 (0.56−0.93)
24	130/1434 (9%)	156/1381 (11%)	0.83 (0.67−1.03)
36	96/1109 (9%)	128/1062 (12%)	0.79 (0.62−0.99)
48	40/465 (9%)	40/401 (10%)	0.90 (0.65−1.23)

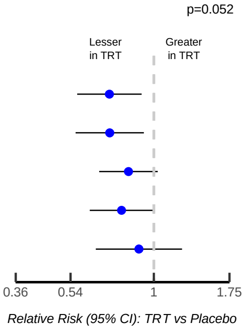

© 2023 Pencina KM et al. *JAMA Network Open.*

Legend. Frequencies and relative risks of incident anemia with the corresponding 95% confidence intervals for prespecified subgroups (age <65, 65 or older; pre-existing CVD or not; baseline testosterone <250 or ≥250; racial group White or Black) of study participants who did not have anemia at baseline are shown by treatment group and time point from four separate 3-way interaction models. The contrast test p value for the difference between treatment groups across all time points within a subpopulation is shown. The p values for the contrast test of interaction that tested if there is a difference in treatment effect between subpopulations across all time points were as follows: Prior CVD vs no prior CVD, p = 0.424; baseline testosterone <250 versus ≥ 250 ng/dL, p = 0.466; age <65 versus ≥65 years, p = 0.329; and race Black versus White, p = 0.445. A compound symmetric covariance matrix was assumed in all models.

© 2023 Pencina KM et al. *JAMA Network Open.*

eFigure 6. Sensitivity Analyses of the Risk of Correction of Anemia Among Participants Who Had Anemia at Baseline

Panel A. Sensitivity analyses of the risk of correction of anemia among participants who had anemia at baseline in which follow-up time was censored 30 days after the last dose of the study medication.

Panel B. Sensitivity analyses of the risk of correction of anemia among participants who had anemia at baseline in which follow-up time was censored 365 days after the last dose of the study medication.

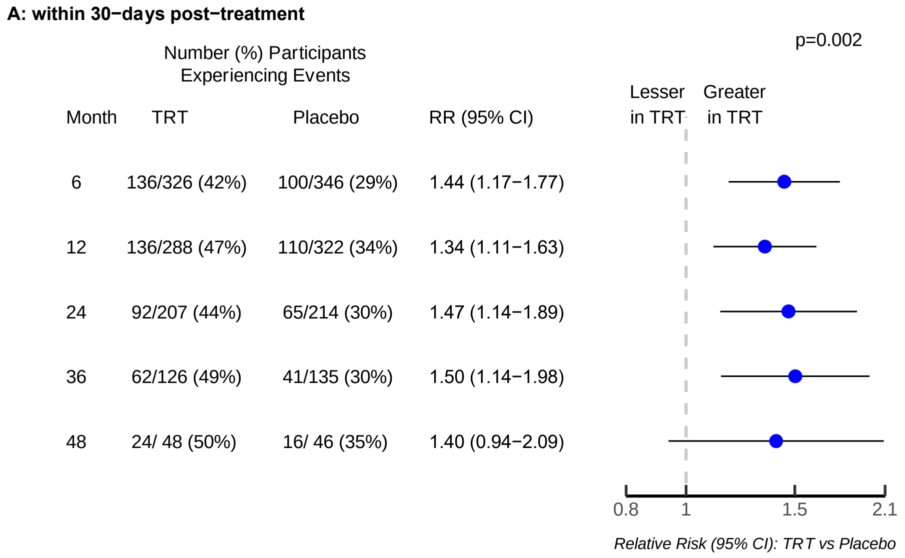

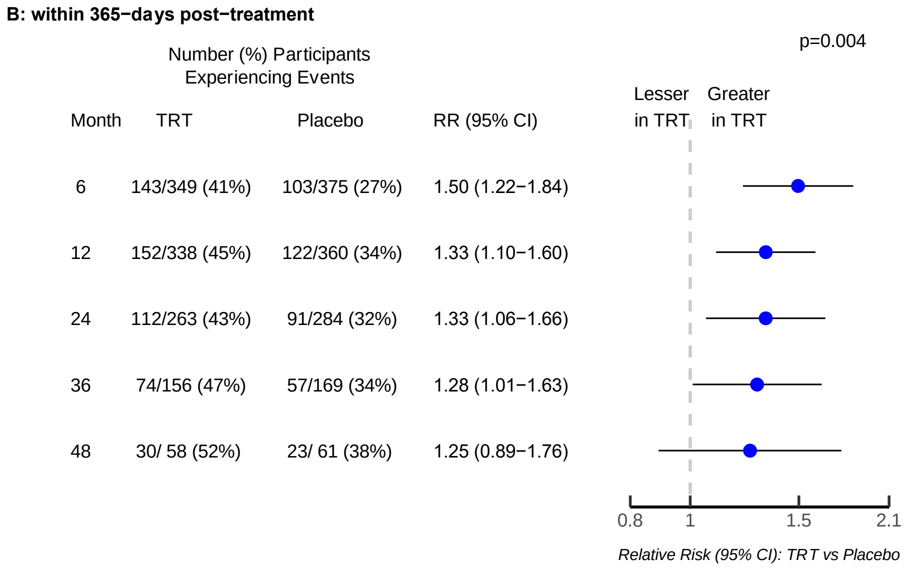

Legend**.** Frequencies and relative risks of correction of anemia in the TRT group relative to placebo group and 95% confidence intervals at each visit in men who had anemia at baseline are shown by treatment group and time point, when follow-up time was censored 30 or 365-days after the last dose of the study medication. The risk ratio of correction of anemia in the TRT versus placebo group was estimated by a repeated measures log- binomial

© 2023 Pencina KM et al. *JAMA Network Open.*

regression with fixed effects for treatment, visit, treatment-visit interaction, and pre-existing cardiovascular disease, and a random per-subject repeated measures effect using an unstructured covariance matrix. The omnibus test p value shown in each figure is a test of the null hypothesis of no difference between TRT and placebo groups across all time points.

© 2023 Pencina KM et al. *JAMA Network Open.*

eFigure 7. Sensitivity Analyses of the Risk of Incident Anemia Among Participants Who Did Not Have Anemia at Baseline

Panel A. Sensitivity analyses of the risk of incident anemia among participants who did not have anemia at baseline in which follow-up time was censored 30 days after the last dose of the study medication.

Panel B. Sensitivity analyses of the risk of incident anemia among participants who did not have anemia at baseline in which follow-up time was censored 365 days after the last dose of the study medication.

A: within 30−days post−treatment

Number (%) Participants Experiencing Events

Month	TRT	Placebo	RR (95% CI)
6	113/1874 (6%)	173/1852 (9%)	0.65 (0.52−0.81)
12	97/1659 (6%)	140/1665 (8%)	0.70 (0.54−0.90)
24	120/1259 (10%)	144/1204 (12%)	0.83 (0.66−1.03)
36	69/795 (9%)	106/766 (14%)	0.75 (0.58−0.98)
48	21/266 (8%)	29/242 (12%)	0.69 (0.46−1.05)

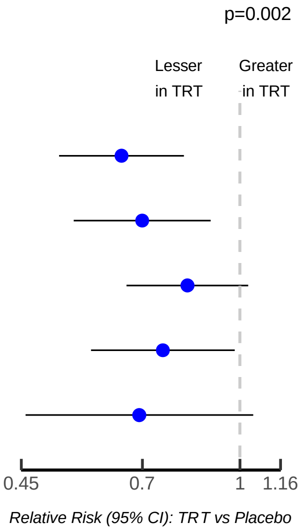

B: within 365−days post−treatment

Number (%) Participants Experiencing Events

Month	TRT	Placebo	RR (95% CI)
6	132/1986 (7%)	197/1952 (10%)	0.66 (0.54−0.82)
12	127/1923 (7%)	168/1888 (9%)	0.74 (0.59−0.92)
24	151/1607 (9%)	181/1559 (12%)	0.83 (0.68−1.01)
36	95/963 (10%)	128/944 (14%)	0.83 (0.66−1.04)
48	28/339 (8%)	37/307 (12%)	0.75 (0.52−1.08)

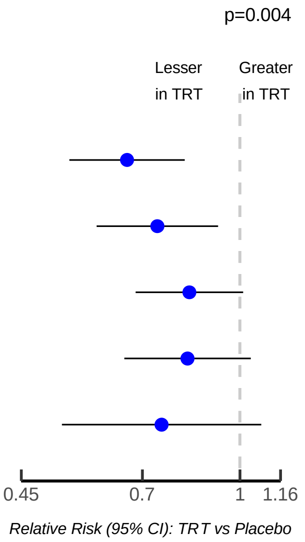

Legend**.** Frequencies and relative risks of incident anemia in the TRT group relative to placebo group and 95% confidence intervals at each visit in men did not have anemia at baseline are shown by treatment group and time point, when follow-up time was censored 30 or 365-days after the last dose of the study medication. The risk ratio of incident anemia in the TRT versus placebo group was estimated by a repeated measures log- binomial regression

© 2023 Pencina KM et al. *JAMA Network Open.*

with fixed effects for treatment, visit, treatment-visit interaction, and pre-existing cardiovascular disease, and a random per-subject repeated measures effect using an unstructured covariance matrix. The omnibus test p value shown in each figure is a test of the null hypothesis of no difference between TRT and placebo groups across all time points.

© 2023 Pencina KM et al. *JAMA Network Open.*

eTable 1. Change From Baseline in Red Cell Counts and Hematocrit Levels

Test	Population	Month	Study Arm	N	Baseline Mean (SD)	Visit Mean (SD)	Change from baseline		Treatment Difference		P value
							LS Mean	95% CI	LS Mean	95% CI	
Hematocrit (%)	Anemia	6	Testosterone	340	36.4 (2.4)	38.4 (3.7)	2.1	(1.7,2.4)	1.3	(0.8,1.8)	<0.001
			Placebo	359	36.3 (2.4)	37.0 (3.6)	0.8	(0.4,1.1)			
		12	Testosterone	271	36.4 (2.4)	38.4 (3.8)	1.9	(1.5,2.4)	1.2	(0.6,1.8)	
			Placebo	296	36.3 (2.3)	37.0 (3.9)	0.8	(0.4,1.2)			
		24	Testosterone	184	36.4 (2.4)	38.3 (4.4)	1.6	(1.0,2.2)	1.0	(0.1,1.8)	
			Placebo	179	36.4 (2.3)	37.2 (4.1)	0.7	(0.1,1.2)			
		36	Testosterone	136	36.5 (2.6)	38.5 (4.4)	1.6	(1.0,2.3)	0.9	(-0.1,1.8)	
			Placebo	131	36.5 (2.0)	37.0 (4.1)	0.8	(0.1,1.4)			
		48	Testosterone	53	36.7 (2.4)	39.0 (4.7)	2.1	(1.2,3.1)	-0.2	(-1.6,1.3)	
			Placebo	48	36.7 (2.0)	38.8 (4.4)	2.3	(1.3,3.3)			
	No Anemia	6	Testosterone	1927	42.9 (3.1)	44.7 (4.2)	1.8	(1.6,1.9)	1.7	(1.5,1.9)	<0.001
			Placebo	1884	42.9 (3.0)	43.0 (3.6)	0.1	(-0.1,0.2)			
		12	Testosterone	1568	43.0 (3.0)	44.3 (4.2)	1.3	(1.2,1.5)	1.6	(1.4,1.9)	
			Placebo	1555	42.9 (3.0)	42.6 (3.6)	-0.3	(-0.5,-0.2)			
		24	Testosterone	1129	43.0 (3.0)	43.4 (4.4)	0.4	(0.2,0.5)	1.4	(1.1,1.7)	
			Placebo	1082	43.0 (3.0)	41.9 (3.8)	-1.1	(-1.3,-0.9)			
		36	Testosterone	830	43.0 (2.9)	43.3 (4.3)	0.2	(-0.0,0.4)	1.2	(0.8,1.5)	
			Placebo	810	43.0 (3.0)	42.0 (3.9)	-0.9	(-1.2,-0.7)			
		48	Testosterone	252	43.1 (3.0)	43.7 (5.2)	0.5	(0.1,1.0)	0.9	(0.3,1.5)	
			Placebo	219	43.3 (3.0)	42.9 (3.7)	-0.4	(-0.8,0.0)			
RBC Count ($x10^{12}$/L)	Anemia	6	Testosterone	341	4.2 (0.4)	4.4 (0.5)	0.2	(0.2,0.3)	0.2	(0.1,0.2)	<0.001
			Placebo	360	4.2 (0.4)	4.2 (0.5)	0.1	(0.0,0.1)			
		12	Testosterone	271	4.2 (0.4)	4.4 (0.5)	0.2	(0.2,0.3)	0.1	(0.1,0.2)	
			Placebo	297	4.2 (0.4)	4.2 (0.5)	0.1	(0.0,0.1)			
		24	Testosterone	185	4.2 (0.4)	4.4 (0.5)	0.2	(0.1,0.3)	0.1	(0.0,0.2)	
			Placebo	179	4.2 (0.4)	4.3 (0.5)	0.1	(0.0,0.2)			
		36	Testosterone	136	4.2 (0.4)	4.4 (0.5)	0.2	(0.1,0.2)	0.1	(0.0,0.2)	
			Placebo	131	4.1 (0.4)	4.2 (0.6)	0.1	(0.0,0.1)			
		48	Testosterone	53	4.2 (0.4)	4.4 (0.6)	0.2	(0.1,0.3)	-0.0	(-0.2,0.1)	
			Placebo	48	4.2 (0.4)	4.4 (0.6)	0.2	(0.1,0.3)			
	No Anemia	6	Testosterone	1934	4.8 (0.4)	5.0 (0.5)	0.2	(0.2,0.2)	0.2	(0.2,0.2)	<0.001
			Placebo	1892	4.8 (0.4)	4.8 (0.4)	-0.0	(-0.0,-0.0)			
		12	Testosterone	1573	4.8 (0.4)	5.0 (0.5)	0.2	(0.2,0.2)	0.2	(0.2,0.2)	
			Placebo	1563	4.8 (0.4)	4.8 (0.4)	-0.0	(-0.0,0.0)			

© 2023 Pencina KM et al. *JAMA Network Open.*

		24	Testosterone	1131	4.8 (0.4)	4.9 (0.5)	0.1	(0.1,0.1)	0.2	(0.1,0.2)	
			Placebo	1088	4.8 (0.4)	4.7 (0.4)	-0.1	(-0.1,-0.1)			
		36	Testosterone	835	4.8 (0.4)	4.9 (0.5)	0.0	(0.0,0.1)	0.1	(0.1,0.1)	
			Placebo	811	4.8 (0.4)	4.7 (0.4)	-0.1	(-0.1,-0.0)			
		48	Testosterone	253	4.8 (0.4)	4.9 (0.5)	0.0	(-0.0,0.0)	0.1	(0.0,0.2)	
			Placebo	220	4.8 (0.4)	4.7 (0.4)	-0.1	(-0.1,-0.1)			

Legend Means and standard deviations (SD) are presented for baseline and each visit for subjects with a measurement at the visit. Least Square Means (LS Means) estimates of change from baseline (95% confidence interval (CI)) for each group and the difference between TRT and placebo at each visit are derived from a linear mixed effects model controlling for baseline value and pre-existing CVD, assuming an unstructured covariance matrix.

© 2023 Pencina KM et al. *JAMA Network Open.*

eTable 2. Changes in Red Blood Cell Indices

Test	Population	Month	Study Arm	N	Baseline Mean (SD)	Visit Mean (SD)	Change from baseline		Treatment Difference		P value
							LS Mean	95% CI	LS Mean	95% CI	
MCV (fL)	Anemia	6	Testosterone	340	87.0 (7.0)	87.0 (6.9)	-0.1	(-0.5,0.4)	-0.5	(-1.1,0.2)	0.541
			Placebo	359	87.8 (6.5)	88.1 (6.5)	0.4	(-0.1,0.8)			
		12	Testosterone	271	87.0 (6.8)	87.1 (6.7)	0.1	(-0.4,0.5)	0.1	(-0.6,0.7)	
			Placebo	296	87.5 (6.6)	87.4 (6.7)	0.0	(-0.4,0.5)			
		24	Testosterone	184	87.3 (6.5)	87.3 (6.8)	-0.0	(-0.6,0.6)	0.3	(-0.5,1.2)	
			Placebo	179	88.1 (6.2)	87.6 (6.3)	-0.4	(-1.0,0.3)			
		36	Testosterone	136	87.2 (6.7)	87.3 (7.1)	0.3	(-0.4,1.0)	-0.1	(-1.1,1.0)	
			Placebo	131	88.5 (6.6)	88.4 (6.6)	0.4	(-0.4,1.1)			
		48	Testosterone	53	87.3 (6.4)	88.2 (6.7)	1.6	(0.6,2.6)	0.1	(-1.4,1.5)	
			Placebo	48	87.7 (6.8)	88.9 (7.4)	1.6	(0.5,2.6)			
	No Anemia	6	Testosterone	1927	89.4 (4.8)	89.7 (5.4)	0.2	(0.1,0.4)	-0.3	(-0.5,-0.1)	0.019
			Placebo	1884	89.8 (5.0)	90.3 (5.2)	0.5	(0.4,0.7)			
		12	Testosterone	1568	89.4 (4.9)	89.0 (5.2)	-0.5	(-0.6,-0.3)	-0.1	(-0.3,0.1)	
			Placebo	1555	89.9 (5.0)	89.5 (5.1)	-0.4	(-0.5,-0.2)			
		24	Testosterone	1129	89.5 (4.8)	88.7 (5.1)	-0.8	(-1.0,-0.6)	0.0	(-0.2,0.3)	
			Placebo	1082	90.0 (4.9)	89.0 (5.0)	-0.9	(-1.1,-0.7)			
		36	Testosterone	830	89.5 (4.7)	89.1 (5.4)	-0.3	(-0.5,-0.0)	0.4	(0.0,0.7)	
			Placebo	810	89.9 (4.8)	89.2 (5.1)	-0.6	(-0.9,-0.4)			
		48	Testosterone	252	89.3 (4.5)	89.9 (5.5)	0.9	(0.5,1.4)	-0.1	(-0.8,0.5)	
			Placebo	219	89.9 (4.7)	90.8 (5.6)	1.1	(0.6,1.6)			
MCH (pg)	Anemia	6	Testosterone	341	28.4 (2.8)	28.4 (2.7)	-0.1	(-0.2,0.1)	-0.3	(-0.5,-0.0)	0.213
			Placebo	360	28.8 (2.5)	29.0 (2.5)	0.2	(0.1,0.4)			
		12	Testosterone	271	28.4 (2.7)	28.6 (2.7)	0.2	(0.0,0.4)	-0.1	(-0.4,0.1)	
			Placebo	297	28.7 (2.5)	29.1 (2.6)	0.4	(0.2,0.6)			
		24	Testosterone	185	28.5 (2.6)	29.0 (2.5)	0.4	(0.2,0.7)	-0.0	(-0.4,0.3)	
			Placebo	179	28.8 (2.5)	29.3 (2.5)	0.5	(0.2,0.7)			
		36	Testosterone	136	28.4 (2.7)	28.9 (2.9)	0.4	(0.1,0.7)	0.2	(-0.3,0.6)	
			Placebo	131	29.0 (2.5)	29.2 (2.5)	0.3	(-0.1,0.6)			
		48	Testosterone	53	28.1 (2.4)	28.9 (2.5)	0.5	(0.2,0.9)	0.0	(-0.5,0.6)	

© 2023 Pencina KM et al. *JAMA Network Open.*

			Placebo	48	28.7 (2.7)	29.1 (2.8)	0.5	(0.1,0.9)			
	No Anemia	6	Testosterone	1934	30.0 (1.8)	29.8 (2.0)	-0.2	(-0.3,-0.2)	-0.2	(-0.3,-0.1)	<0.001
			Placebo	1892	30.2 (1.8)	30.2 (1.8)	-0.0	(-0.1,0.0)			
		12	Testosterone	1573	30.1 (1.8)	30.1 (2.0)	0.1	(0.0,0.1)	-0.0	(-0.1,0.1)	
			Placebo	1563	30.2 (1.8)	30.3 (1.9)	0.1	(0.0,0.1)			
		24	Testosterone	1131	30.0 (1.8)	30.1 (2.0)	0.0	(-0.1,0.1)	0.0	(-0.1,0.2)	
			Placebo	1088	30.2 (1.7)	30.2 (1.8)	-0.0	(-0.1,0.1)			
		36	Testosterone	835	30.0 (1.7)	29.9 (2.1)	-0.1	(-0.2,-0.0)	0.1	(0.0,0.3)	
			Placebo	811	30.1 (1.7)	30.0 (1.9)	-0.3	(-0.4,-0.2)			
		48	Testosterone	253	29.7 (1.7)	29.7 (2.1)	-0.2	(-0.4,-0.0)	-0.1	(-0.3,0.2)	
			Placebo	220	29.9 (1.6)	30.0 (2.0)	-0.1	(-0.3,0.1)			
MCHC (g/L)	Anemia	6	Testosterone	340	326.5 (15.2)	326.6 (15.0)	-0.2	(-1.7,1.3)	-1.6	(-3.7,0.5)	0.432
			Placebo	359	328.6 (14.5)	329.1 (15.0)	1.4	(-0.1,2.8)			
		12	Testosterone	271	326.1 (14.5)	329.4 (15.7)	2.8	(1.1,4.4)	-1.4	(-3.8,0.9)	
			Placebo	296	328.2 (14.8)	331.7 (15.2)	4.2	(2.6,5.8)			
		24	Testosterone	184	326.5 (14.2)	332.3 (15.3)	5.1	(3.0,7.1)	-2.0	(-4.9,0.9)	
			Placebo	179	327.3 (14.3)	334.7 (14.6)	7.1	(5.0,9.1)			
		36	Testosterone	136	325.4 (14.3)	330.2 (14.5)	3.5	(1.4,5.7)	0.6	(-2.5,3.7)	
			Placebo	131	326.8 (13.0)	330.1 (13.1)	2.9	(0.7,5.1)			
		48	Testosterone	53	323.0 (10.8)	327.9 (13.5)	1.2	(-2.2,4.5)	-1.0	(-5.9,3.9)	
			Placebo	48	327.5 (13.3)	327.9 (16.1)	2.1	(-1.4,5.7)			
	No Anemia	6	Testosterone	1927	336.5 (12.9)	332.9 (14.0)	-3.3	(-3.9,-2.7)	-1.5	(-2.4,-0.7)	0.021
			Placebo	1884	336.6 (13.0)	334.4 (13.8)	-1.8	(-2.4,-1.2)			
		12	Testosterone	1568	336.7 (12.8)	338.7 (13.6)	2.4	(1.8,3.0)	-0.5	(-1.3,0.4)	
			Placebo	1555	336.4 (13.0)	339.0 (13.2)	2.9	(2.3,3.5)			
		24	Testosterone	1129	336.0 (12.2)	339.5 (14.6)	3.4	(2.6,4.1)	0.0	(-1.1,1.1)	
			Placebo	1082	335.5 (12.4)	339.3 (14.6)	3.3	(2.5,4.1)			
		36	Testosterone	830	335.5 (12.3)	335.7 (15.0)	-0.7	(-1.6,0.2)	-0.7	(-2.0,0.6)	
			Placebo	810	335.4 (12.8)	336.4 (14.7)	0.0	(-0.9,1.0)			
		48	Testosterone	252	332.6 (11.3)	330.7 (15.2)	-4.9	(-6.6,-3.2)	-0.6	(-3.1,1.8)	
			Placebo	219	333.1 (12.8)	330.9 (14.5)	-4.3	(-6.1,-2.5)			

Legend Means and standard deviations (SD) are presented for baseline and each visit for subjects with a measurement at the visit. Least Square Means (LS Means) estimates of change from baseline (95% confidence interval (CI)) for each group and the difference between TRT and placebo at each visit are derived from a linear mixed effects model controlling for baseline value and pre-existing CVD, assuming an unstructured covariance matrix.

© 2023 Pencina KM et al. *JAMA Network Open.*

eTable 3. Changes in total testosterone, DHT and estradiol levels

Test	Population	Month	Study Arm	N	Baseline Mean(SD)	Visit Mean(SD)	Change from baseline		Treatment Difference		Pvalue
							LS Mean	95% CI	LS Mean	95% CI	
Testosterone (pg/mL)	Anemia	12	Testosterone	274	211.4 (50.4)	388.2 (334.9)	175.5	(147.0,203.9)	155.8	(116.3,195.3)	<0.001
			Placebo	293	210.8 (49.0)	232.3 (99.7)	19.7	(-7.9,47.3)			
		24	Testosterone	192	210.2 (50.9)	370.2 (269.2)	159.6	(129.7,189.5)	125.7	(82.9,168.5)	
			Placebo	183	210.6 (49.4)	242.7 (124.4)	33.9	(3.3,64.5)			
		36	Testosterone	136	213.2 (48.8)	356.8 (227.8)	143.1	(110.9,175.2)	95.1	(49.4,140.9)	
			Placebo	132	215.7 (45.8)	260.9 (163.6)	47.9	(15.3,80.6)			
		48	Testosterone	54	210.5 (44.5)	291.4 (145.2)	86.8	(50.6,123.0)	29.8	(-22.5,82.1)	
			Placebo	49	206.4 (49.7)	279.6 (202.6)	56.9	(19.1,94.8)			
	No Anemia	12	Testosterone	1588	222.9 (45.9)	430.3 (300.2)	206.5	(195.1,218.0)	172.3	(156.2,188.5)	<0.001
			Placebo	1589	222.0 (47.3)	257.1 (139.6)	34.2	(22.8,45.7)			
		24	Testosterone	1163	223.9 (45.2)	397.8 (246.7)	172.7	(161.4,184.1)	134.0	(117.8,150.2)	
			Placebo	1106	222.9 (46.9)	261.4 (134.0)	38.7	(27.1,50.4)			
		36	Testosterone	841	223.9 (45.2)	390.4 (283.8)	164.3	(149.0,179.7)	112.4	(90.6,134.2)	
			Placebo	825	223.8 (46.2)	275.3 (160.6)	51.9	(36.4,67.4)			
		48	Testosterone	256	222.2 (45.9)	336.3 (201.0)	113.6	(94.4,132.9)	73.8	(45.6,101.9)	
			Placebo	222	223.0 (48.0)	264.7 (105.3)	39.9	(19.3,60.5)			
Dihydrotestosterone (ng/dL)	Anemia	12	Testosterone	284	14.5 (7.3)	55.1 (49.2)	40.4	(36.4,44.3)	38.1	(32.6,43.6)	<0.001
			Placebo	304	14.8 (7.5)	17.1 (10.9)	2.3	(-1.5,6.1)			
		36	Testosterone	149	15.1 (8.2)	48.4 (51.2)	32.6	(26.8,38.3)	29.4	(21.3,37.6)	
			Placebo	145	15.4 (7.3)	18.4 (11.2)	3.1	(-2.7,8.9)			
	No Anemia	12	Testosterone	1658	16.4 (7.9)	61.8 (51.0)	45.2	(43.5,47.0)	43.1	(40.7,45.6)	<0.001
			Placebo	1633	16.6 (8.8)	18.7 (11.2)	2.1	(0.4,3.9)			
		36	Testosterone	907	16.6 (8.0)	57.1 (53.8)	39.9	(37.4,42.3)	35.8	(32.2,39.3)	
			Placebo	877	16.6 (9.2)	20.5 (14.2)	4.1	(1.5,6.6)			
Estradiol (pg/mL)	Anemia	12	Testosterone	279	18.5 (7.3)	27.0 (19.4)	8.4	(6.8,10.0)	8.6	(6.4,10.8)	<0.001
			Placebo	299	18.4 (7.7)	18.4 (7.4)	-0.2	(-1.8,1.3)			
		36	Testosterone	149	18.8 (7.6)	25.0 (13.0)	6.3	(4.5,8.1)	3.6	(1.0,6.2)	
			Placebo	143	19.1 (8.1)	21.5 (11.4)	2.7	(0.8,4.5)			
	No Anemia	12	Testosterone	1637	21.5 (8.2)	30.5 (18.7)	9.0	(8.3,9.6)	9.2	(8.2,10.2)	<0.001
			Placebo	1606	21.4 (8.3)	21.2 (10.5)	-0.2	(-0.9,0.5)			
		36	Testosterone	894	21.4 (8.2)	29.7 (20.3)	8.4	(7.4,9.4)	6.7	(5.3,8.2)	
			Placebo	877	21.3 (8.4)	22.8 (12.5)	1.7	(0.6,2.7)			

Legend Means and standard deviations (SD) are presented for baseline and each visit for subjects with a measurement at the visit. Least Square Means (LS Means) estimates of change from baseline (95% confidence interval (CI)) for each group and the difference between TRT and placebo at each visit are derived from a linear mixed effects model controlling for baseline value and pre-existing CVD, assuming an unstructured covariance matrix. To convert serum total testosterone

© 2023 Pencina KM et al. *JAMA Network Open.*

concentrations in nanograms per deciliter to nanomoles per liter, multiply testosterone concentration in in nanograms per deciliter by 0.0347. To convert estradiol concentrations from picogram per milliliter to picomoles per liter, multiply estradiol concentrations in picogram per milliliter by 3.67. To convert dihydrotestosterone concentrations in nanograms per deciliter to nanomoles per liter, multiply dihydrotestosterone concentrations in nanograms per deciliter by 0.0344.

© 2023 Pencina KM et al. *JAMA Network Open.*

eTable 4. Listing of investigator-reported adverse events in men with anemia and men without anemia

Event	Anemia at baseline			No Anemia at baseline		
	TRT: (N = 390)	Placebo: (N = 425)	P value	TRT: (N = 2203)	Placebo: (N = 2176)	P value
Any adverse event	192 (49.2)	219 (51.5)	0.512	994 (45.1)	945 (43.4)	0.260
Serious adverse event	142 (36.4)	155 (36.5)	0.986	578 (26.2)	542 (24.9)	0.314
Adverse event leading to discontinuation of the study drug	50 (12.8)	51 (12.0)	0.722	194 (8.8)	175 (8.0)	0.363
Pre-specified adverse events of special interest	40 (10.3)	34 (8.0)	0.263	156 (7.1)	133 (6.1)	0.197
Hospitalization for unstable angina	9 (2.3)	11 (2.6)	0.796	35 (1.6)	49 (2.3)	0.110
Non-fatal arrhythmias requiring intervention	26 (6.7)	13 (3.1)	0.016	108 (4.9)	74 (3.4)	0.013
Cardiovascular disease causing syncope	8 (2.1)	9 (2.1)	0.947	19 (0.9)	23 (1.1)	0.509
Transient ischemic attack	1 (0.3)	6 (1.4)	0.074	14 (0.6)	11 (0.5)	0.568
Diabetes mellitus	26 (6.7)	33 (7.8)	0.546	163 (7.4)	180 (8.3)	0.282
COVID-19	20 (5.1)	13 (3.1)	0.134	101 (4.6)	104 (4.8)	0.760
Atrial fibrillation	16 (4.1)	15 (3.5)	0.669	75 (3.4)	48 (2.2)	0.016
Pneumonia	19 (4.9)	13 (3.1)	0.183	45 (2.0)	43 (2.0)	0.875
Acute kidney injury	22 (5.6)	10 (2.4)	0.016	38 (1.7)	30 (1.4)	0.354
Benign prostatic hyperplasia	8 (2.1)	7 (1.6)	0.668	37 (1.7)	39 (1.8)	0.775
Acute respiratory failure	13 (3.3)	8 (1.9)	0.192	39 (1.8)	29 (1.3)	0.242
Urinary retention	10 (2.6)	4 (0.9)	0.075	40 (1.8)	30 (1.4)	0.249

© 2023 Pencina KM et al. *JAMA Network Open.*

Event	Anemia at baseline			No Anemia at baseline		
	**TRT: (N = 390)**	**Placebo: (N = 425)**	**P value**	**TRT: (N = 2203)**	**Placebo: (N = 2176)**	**P value**
Cellulitis	3 (0.8)	14 (3.3)	0.012	32 (1.5)	32 (1.5)	0.960
Cardiac failure congestive	14 (3.6)	11 (2.6)	0.408	20 (0.9)	30 (1.4)	0.143

Legend. P values were calculated using a chi-square test.

© 2023 Pencina KM et al. *JAMA Network Open.*
