# Supplementary material for: Efficacy of Testosterone Replacement Therapy in Correcting Anemia in Men With Hypogonadism: A Randomized Clinical Trial
Source: JAMA Netw Open. 2023 Oct 27;6(10):e2340030. doi: 10.1001/jamanetworkopen.2023.40030 (PMC10611996; doi:10.1001/jamanetworkopen.2023.40030)
Supplement: Supplement 3. — Data Sharing Statement [file jamanetwopen-e2340030-s003.pdf]

Data Sharing Statement

Pencina. Efficacy of Testosterone Replacement Therapy in Correcting Anemia in Men with Hypogonadism. *JAMA Netw Open*. Published October 27, 2023.
doi:10.1001/jamanetworkopen.2023.40030

Data
Data available: Yes
Data types: Deidentified participant data
How to access data: Deidentified data will be made available upon submission of a written request to TRAVERSE Anemia Study Committee by writing to sbhasin@bwh.harvard.edu
When available: With publication

Supporting Documents
Document types: None

Additional Information
Who can access the data: The data will be made available to researchers for noncommercial purposes. A materials transfer agreement and a DUA will be put in place before data transfer.
Types of analyses: The data will be made available to researchers for noncommercial research or educational purposes if similar analyses have not been previously conducted or planned and there are no competing requests.
Mechanisms of data availability: The data will be made available with a with a signed data access agreement and / or a material transfer agreement.
Any additional restrictions: The data may not be made available for commercial purposes or for analyses or research that is already underway or planned, or if there is a competing preceding request for the same set of data and analyses.
